# Processing of acceleration and dive data on‐board satellite relay tags to investigate diving and foraging behaviour in free‐ranging marine predators

**DOI:** 10.1111/2041-210X.12845

**Published:** 2017-07-27

**Authors:** Sam L. Cox, Florian Orgeret, Mathieu Gesta, Charles Rodde, Isaac Heizer, Henri Weimerskirch, Christophe Guinet

**Affiliations:** ^1^ Centre d'Etudes Biologique de Chizé U.M.R. 7372 – CNRS & Universitié de La Rochelle Villiers‐en‐Bois France; ^2^ Wildlife Computers Redmond WA USA

**Keywords:** accelerometers, animal biotelemetry, Argos, data abstraction, *Mirounga leonina*, prey catch attempts, satellite data relaying, southern elephant seal, swimming effort

## Abstract

1. Biologging technologies are changing the way in which the marine environment is observed and monitored. However, because device retrieval is typically required to access the high‐resolution data they collect, their use is generally restricted to those animals that predictably return to land. Data abstraction and transmission techniques aim to address this, although currently these are limited in scope and do not incorporate, for example, acceleration measurements which can quantify animal behaviours and movement patterns over fine‐scales.
2. In this study, we present a new method for the collection, abstraction and transmission of accelerometer data from free‐ranging marine predators via the Argos satellite system. We test run the technique on 20 juvenile southern elephant seals *Mirounga leonina* from the Kerguelen Islands during their first months at sea following weaning. Using retrieved archival data from nine individuals that returned to the colony, we compare and validate abstracted transmissions against outputs from established accelerometer processing procedures.
3. Abstracted transmissions included estimates, across five segments of a dive profile, of time spent in prey catch attempt (PrCA) behaviours, swimming effort and pitch. These were then summarised and compared to archival outputs across three dive phases: descent, bottom and ascent. Correlations between the two datasets were variable but generally good (dependent on dive phase, marginal *R* ^2^ values of between .45 and .6 to >.9) and consistent between individuals. Transmitted estimates of PrCA behaviours and swimming effort were positively biased to those from archival processing.
4. Data from this study represent some of the first remotely transmitted quantifications from accelerometers. The methods presented and analysed can be used to provide novel insight towards the behaviours and movements of free‐ranging marine predators, such as juvenile southern elephant seals, from whom logger retrieval is challenging. Future applications could however benefit from some adaption, particularly to reduce positive bias in transmitted PrCA behaviours and swimming effort, for which this study provides useful insight.

## 1 INTRODUCTION

Observing and studying animals in their natural environment is challenging, particularly for those marine species that spend long periods of time at sea ranging over vast distances. The field of biologging aims to address this through the animal attachment of miniaturised devices, capable of recording and/or relaying measures of an individual's movement, physiology and/or surrounding environment (Cooke et al., 2004; Fedak, Lovell, McConnell, & Hunter, 2002; Rutz & Hays, 2009). These data can then be quantified via a variety of sophisticated analyses, to provide information on an animal's at‐sea ecology at an unprecedented level of detail and range of spatio‐temporal scales (Carter, Bennett, Embling, Hosegood, & Russell, 2016; Heerah, Hindell, Guinet, & Charrassin, 2014; Shepard et al., 2008; Viviant, Trites, Rosen, Monestiez, & Guinet, 2010).

The advent of accelerometers in biologging studies has been key to recent developments in the field (Brown, Kays, Wikelski, Wilson, & Klimley, 2013; Wilson, Shepard, & Liebsch, 2008). Since these devices measure both the orientation and movement dynamics of an animal, a range of previously unobservable behavioural metrics can now be described and analysed (Brown et al., 2013; Shepard et al., 2008). In particular, such information has revolutionised our ability to study the behaviours of diving predators, which often spend extended periods of time underwater and are thus hard to monitor. For example, prey catch attempt (PrCA) behaviours can be identified from peaks in acceleration indicative of rapid head movements (Gallon et al., 2013; Naito, Bornemann, Takahashi, McIntyre, & Plotz, 2010; Suzuki, Naito, Folkow, Miyazaki, & Blix, 2009; Viviant et al., 2010; Volpov, Hoskins, et al., 2015), whilst proxies of energetic expenditure (e.g. swimming effort) can be calculated by isolating dynamic movement rates (Jeanniard‐du‐Dot, Guinet, Arnould, Speakman, & Trites, 2017; Sato, Mitani, Cameron, Siniff, & Naito, 2003; Volpov, Rosen, Trites, & Arnould, 2015). The latter of these can additionally be used in tandem with estimations of pitch and vertical speed to give a relative measure of body condition (Aoki et al., 2011; Richard et al., 2014). However, obtaining the data collected by accelerometers generally requires device retrieval. In some instances, remote downloading via radio transmitters and mobile phone technology may be possible, although typically this still requires an individual to return to within a few kilometres of a set location (Brown et al., 2013). As such, whilst animals that return to land (e.g. central place foragers such as breeding pinnipeds and seabirds) are well represented in the literature, there is a paucity of studies investigating the at‐sea behaviours and movements of free‐ranging individuals that remain at sea for extended periods (Hart & Hyrenbach, 2009; Hazen et al., 2012; McIntyre, 2014). In particular, information is missing from immature and juvenile stages (where a large proportion of individuals die within the first few months at sea and those that do survive often remain offshore for long periods of time and lose their loggers before returning to land), alongside seal species associated with sea‐ice marginal zones—which are almost impossible to recapture for device retrieval (e.g. harp *Pagophilus groenlandicus*, hooded *Cystophora cristata* and leopard *Hydrurga leptonyx* seals).

Satellite data relay tags (using the Argos satellite system) can remotely transmit data collected by archival loggers, negating the need to retrieve devices. As such, their use has the potential to address current gaps in the literature with regards to the use of accelerometers. However, the amount of data that can be successfully communicated is limited due to, for example, the battery life of a device, an individual's behaviour (short surfacing periods that cut transmission), software specifications (processing power) and satellite platform (e.g. Argos transmission message lengths must currently be between 32 and 248 [or 256] bits; CLS, 2016; Fedak et al., 2002). Subsequently, obtaining information equal to that acquired via tag retrieval is challenging. Data abstraction, performed via pre‐transmission on‐board processing, can be used to produce simplified representations of large volumes of data, thus aiding the relaying of information via satellite systems (Fedak et al., 2002; Photopoulou, Fedak, Matthiopoulos, McConnell, & Lovell, 2015). However, to accommodate extended deployment durations and maximise data acquisition, power requirements must be minimised. Previous implementations have included the execution of simple algorithms capable of extracting basic behavioural metrics such as the maximum depth and duration of a dive, alongside the following surface interval. Extending beyond this, broken‐stick models (BSMs; Fedak, Lovell, & Grant, 2001; Photopoulou, Lovell, Fedak, Thomas, & Matthiopoulos, 2015) can identify key inflection points along depth profiles, providing information on a dive's shape. However, despite the unparalleled information accelerometers can provide on an individual's movement patterns, currently there exists no method to abstract and transmit their data. This is likely due to the huge amount of data produced and predominantly complicated processing procedures required for analysis.

In this study, we present a new method for collecting, abstracting and transmitting accelerometer data from free‐ranging marine predators via the Argos satellite system. We test run the technique on juvenile southern elephant seals *Mirounga leonina* from the Kerguelen Islands during their first months at sea after weaning, and validate the method using established accelerometer processing techniques applied to archival data recovered from nine individuals that returned to the colony. Few studies have investigated how the early foraging behaviours of these animals develop, despite the typically high mortality rates experienced during these times (McMahon & Burton, 2005; Pistorius & Bester, 2002). Information from accelerometers can help address this, and so, by selecting this free‐ranging marine predator to test run our method, we also aim to obtain data that can be used in later studies to investigate ontogenetic changes in at‐sea behaviours and differences between individuals that may impact survival. An overview and assessment of how simplified methods can be used to process accelerometer data into short behavioural summaries suitable for transmission is provided, so that such approaches will be applied in future studies using satellite relay systems to investigate the dive and foraging behaviours of free‐ranging marine species.

## 2 MATERIALS AND METHODS

### 2.1 Tag deployment

**Figure 1:**
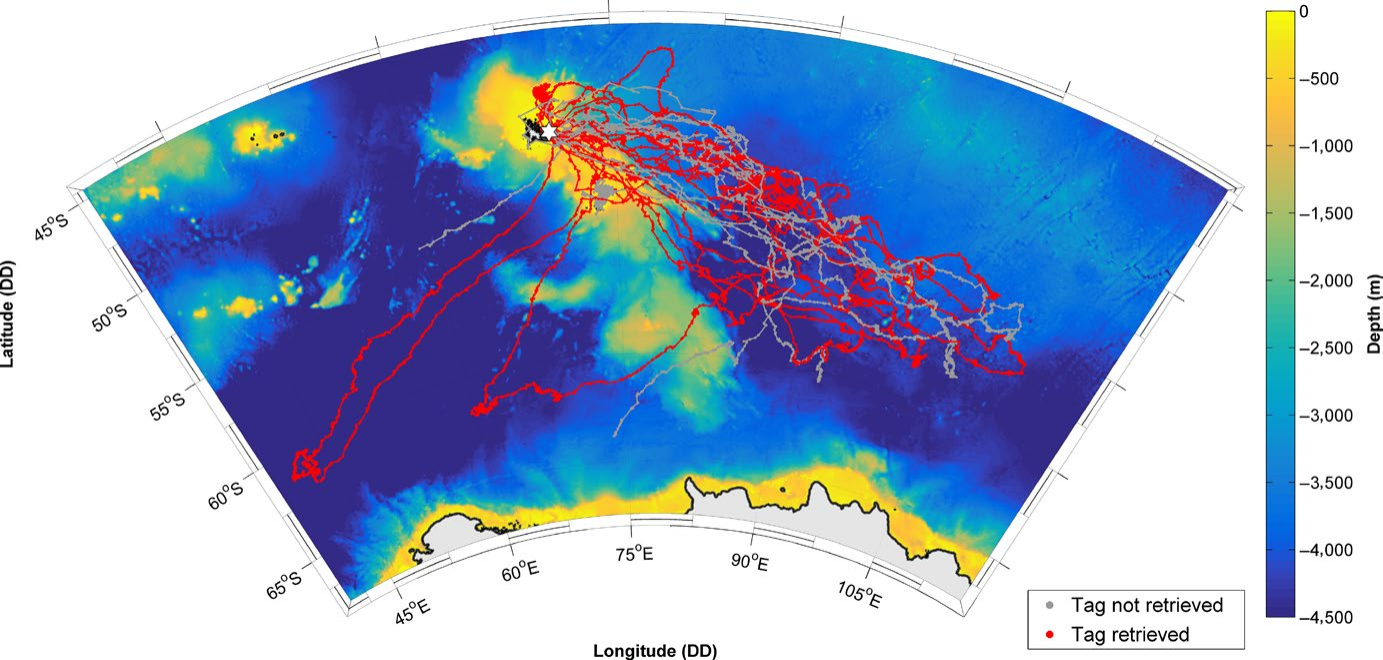
Recorded tracks of each individual. In grey, those from which tags were not retrieved and in red, those from which tags were retrieved. The deployment site at Kerguelen Islands is indicated by the white star

Fieldwork was conducted at Kerguelen Islands (49°20′S 70°20′E; Figure 1), Sub‐Antarctic during November/December 2014. A total of 20 (10 female and 10 male) weaned juvenile southern elephant seals (age ~8–10 weeks; mean mass 79.9 ± 17.7 kg, mean length 39.1 ± 10.6 cm; ±*SD*; Supplementary material S.1) were equipped with a custom designed Argos relay satellite tag (SCOUT‐DSA‐296 tag, Wildlife Computers, hereafter “DSA” tag). This weighed less than 1% of the mean mass of a pup, and was of similar design to those from investigative studies demonstrating such devices do not adversely impact mass gain and survival of equipped individuals (McMahon, Field, Bradshaw, White, & Hindell, 2008). Animals were captured and anaesthetised (80% of tagged individuals) using 0.2–0.3 ml of a 1:1 combination of tiletamine and zolazepam (Zoletil 100), injected intravenously. After cleaning the fur with acetone, the DSA tag was attached to the fur of the top of the head of a pup using quick‐setting epoxy (~10 min; Araldite AW 2101, Ciba). All fieldwork activities were approved by the Comité Environnement et le Préfet des Terre Australes et Antarctiques Françaises**.**

### 2.2 Tag specifications

The DSA tag measured 86 × 85 × 29 mm and weighed 192 g. It consisted of an Argos transmitter, pressure sensor, tri‐axial accelerometer and wet‐dry sensor. To conserve battery life and maximise deployment periods, the pressure sensor and tri‐axial accelerometer were programmed to function intermittently, such that one complete dive was sampled every ~2.25 hr. To achieve this, the tag would cease recording for 2 hr from the timestamp of the end of a dive. A dive was defined as a sub‐surface period lasting longer than 60 s, during which the maximum recorded depth exceeded 15 m. The beginning and end of a dive was determined via the tag's wet‐dry sensor. During recording periods, the pressure sensor recorded at a rate of 1 Hz (resolution of 0.5 m and accuracy of ±1 m + 1% of a reading) and the tri‐axial accelerometer at 16 Hz (resolution of 0.05 m/s^2^). Data corresponding to sampled dives were archived and then processed on‐board to create a “per dive” summary (see details below), which was later transmitted via the Argos satellite system while the tag was at the surface. In addition, the tag also recorded the time from the end of a sampled dive (when the wet‐dry sensor first registered as dry following a dive) to the subsequent first depth measurement that exceeded 10 m to give the surface interval. The tag transmitted each dive summary a maximum of 12 times, with a minimum interval of 45 min between each uplink attempt. At least one Argos location was obtained each day from resulting transmissions.

### 2.3 Pre‐transmission and archival processing methods

Pre‐transmission data abstraction involved a series of tailored processing algorithms, developed specifically for the low power computing environment of the satellite data relay tag from established techniques typically used (and validated) for the processing of archived accelerometer data from pinnipeds (e.g. Gallon et al., 2013; Guinet et al., 2014; Jeanniard‐du‐Dot, Trites, Arnould, Speakman, & Guinet, 2016; Jouma'a et al., 2016; Richard et al., 2014; Viviant et al., 2010; Volpov, Hoskins, et al., 2015). In some instances, this required considerable modification of the original established processing algorithms, alongside the use of set thresholds. Because we did not have prior access to juvenile southern elephant seal accelerometer data (nor that from other small pinnipeds), these were developed, using outputs from adult female southern elephant seals (e.g. using data from Richard et al., 2014; Vacquie‐Garcia et al., 2015). Post‐hoc validation was then completed by comparing transmitted data summaries to outputs from original established accelerometer processing methods applied to archival data from nine individuals that returned to the colony. Details of the two techniques are outlined below.

#### 2.3.1 Dive segmentation: BSMs

The start of a dive was identified on‐board the DSA tag using a wet‐dry sensor. Dive events were identified from the archival data as periods when the tag exceeded a depth of 5 m (in place of 0 m to avoid interference from near surface measurement noise) for at least 60 s. To account for a small amount of sensor drift with time, prior to this, recursive filtering and smoothing across set quantiles was used to apply a zero offset correction to the time series of archival depth measurements (see Luque & Fried, 2010). No depth correction was performed on‐board the DSA tag. In both pre‐transmission data abstraction and post‐hoc archival processing, each dive was then split into five segments by identifying the four most characteristic inflection points of the profile (inclusive of the maximum depth) via BSMs (Fedak et al., 2001). From these, the total duration and initial and final depths of each segment were recorded.

#### 2.3.2 Detection of PrCA behaviours

The time spent in PrCA behaviours was summed across each segment in both pre‐transmission data abstraction and post‐hoc archival processing. This involved the identification of rapid body and head movements, which have been found to be good indicators of PrCA behaviours across a range of marine species (Carroll, Slip, Jonsen, & Harcourt, 2014; Viviant et al., 2010; Volpov, Hoskins, et al., 2015; Ydesen et al., 2014).

##### Pre‐transmission abstraction method

Using accelerations measured along the three axes, magnitude in acceleration mag*A* was calculated as:

(1) $$\text{mag}A_{i}\;=\;\sqrt{x_{i}^{2}+y_{i}^{2}+z_{i}^{2}}$$

Change in mag*A* over 1s was then determined by summing the absolute values of the finite backward differences of 16 successive mag*A* values, resulting in a per second var*S* value, that gave a measure of change comparable to the variance of a 1s sampling period (but required less computing power to calculate):

(2) $$\text{var}S=\sum_{i=2}^{16}\text{abs}\left(\text{mag}A_{i}-\text{mag}A_{i-1}\right)$$

A running average, across a window of 11 s, was then applied to the time series vector of var*S* values to produce a per second average of change in acceleration as var*A*. A PrCA behaviour occurred when var*S*_*i*_ ≥ var*A*_*i*_ + thres*V*, where thres*V* is a user selectable threshold. For this study, a value of 5 m/s^2^ was used (see Supplementary material S.2).

##### Post hoc established archival method

**Figure 2:**
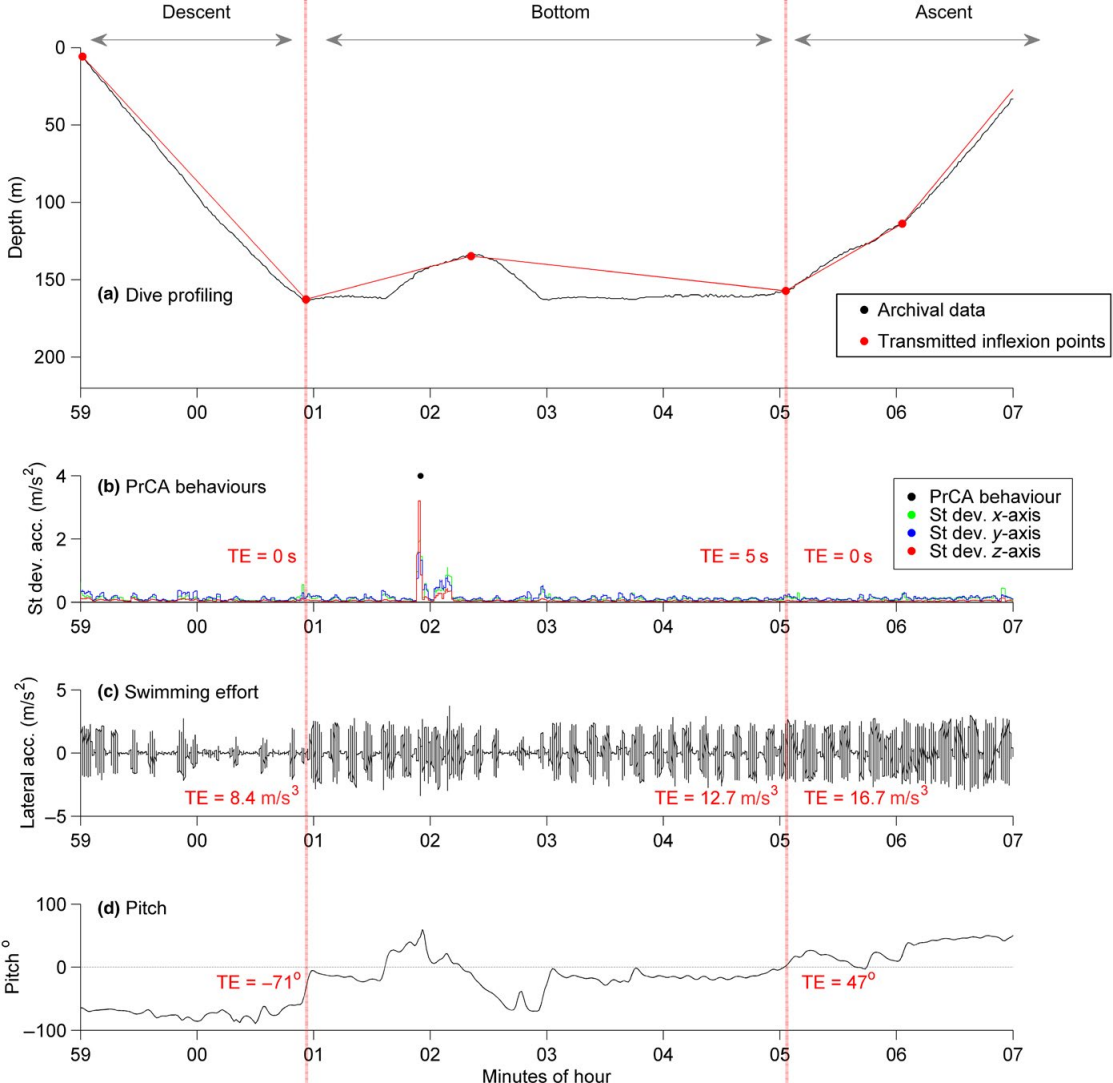
Overview of DSA tag data measurements from one example dive, illustrating the high resolution archives prior to summarising for comparison to segment abstractions from transmitted data. Red dotted partitions from left to right correspond to descent, bottom and ascent dive phases. From top to bottom: (a) depth measurements from archival data in black and reconstructions from transmitted inflection points in red (determined via brokenstick models (BSMs), and used in estimations of inflection depths, phase durations and vertical speeds), (b) standard deviations in acceleration from archival data along the three axes (green = *x*, blue = *y* and *z*  =  red)—black markers show identified prey catch attempt (PrCA) behaviours, (c) filtered lateral (*y*) accelerations from archival data showing stroke amplitudes and rates (used to estimate swimming effort) and (d) pitches from archival data. Red text (b:d) indicates corresponding transmitted estimates (TEs) for each dive phase

Dynamic accelerations (from flipper strokes and rapid head movements) were isolated from gravitational forces along each of the three axes, using a third‐order high‐pass digital Butterworth filter of 2.64 Hz (Guinet et al., 2014; Viviant et al., 2010). For each axis, standard deviations in acceleration were calculated over a moving window of 1.5 s which were then grouped into two, “low” and “high,” states using *k*‐means clustering. Prey catch attempt behaviours occurred when the moving window standard deviation values from all three axes were in a “high” state (Figure 2; Guinet et al., 2014; Vacquie‐Garcia et al., 2015; Viviant et al., 2010).

#### 2.3.3 Swimming effort

Estimations of swimming effort were summed across each segment in both pre‐transmission data abstraction and post‐hoc archival processing. These were then divided by the total duration of a segment to give a per second average (Jouma'a et al., 2016).

##### Pre‐transmission abstraction method

Accelerations from the *y* (lateral) axis were high‐pass filtered using a second‐order IIR Butterworth filter with a 3 dB cut‐off set at 0.2 Hz. This threshold had no upper limit (as is typically used; Jouma'a et al., 2016; Richard et al., 2014) to allow for an expected upwards shift in the frequency range of stroke movements with changes in juvenile body condition and size (Aoki et al., 2011; Richard et al., 2014; Sato et al., 2007). The absolute total of these accelerations was then taken as the swimming effort.

##### Post hoc established archival method

Swimming effort was estimated by isolating and summing flipper stroke rates and movement intensities (Jouma'a et al., 2016; Richard et al., 2014; Sato et al., 2003). For each individual, accelerations from the lateral (*y*) axis were filtered using a third‐order band pass Butterworth filter, centred on the second peak in power intensity as identified from the power spectral density of the signal (between 0.49  and 2.05 Hz; Supplementary material S.3). Swimming effort was then calculated as the absolute sum of peaks (and troughs) of flipper stroke accelerations with absolute amplitudes/intensities of at least 0.2 m/s^2^ (Supplementary material S.4; Richard et al., 2014).

#### 2.3.4 Pitch

Pitch was averaged across the first and last segments in pre‐transmission data abstraction (because the descent and ascent phases of a dive should correspond to these respectively), and across all segments in post hoc archival processing. In both scenarios, each of the three accelerometer axes were filtered using a low‐pass filter of 0.2 Hz. Pitch was then calculated following Tuck (2007) as:

(3) $$\text{pitch}=\text{arctan}\left(\frac{x}{\sqrt{\left(y^{2}+z^{2}\right)}}\right)$$

### 2.4 Comparative analyses between transmitted abstracted and retrieved archival data

Because behavioural differences across descent, bottom and ascent phases may impact the performance of on‐board processing algorithms (and thus quality of transmitted data), comparative analyses between the abstracted and retrieved archival data were conducted separately for each of these phases (see Supplementary material S.5 for details on how dive phases were assigned). For both datasets, all data corresponding to a particular dive phase were either averaged or summed to provide a single comparative value (see details in Supplementary material S.6—which also outlines how archival and transmitted datasets were matched alongside pre‐analytical data cleaning protocols). Of note, for pitch estimates, descent and ascent values from transmitted datasets were based upon the first and last segments of a dive, whilst archival values comprised data from all segments corresponding to a dive phase. This was so we could assess if pitches from the first and last segments of a dive were representative of those from the entire descent and ascent phases.

To allow for, and assess inter‐individual differences in the performance of data abstraction algorithms, linear regressions were fitted using a mixed modelling framework from the nlme package (Pinheiro & Bates, 2014) in r. Across all analyses, transmitted data were fitted as the response variable, and retrieved archival data as the explanatory variable. To validate the on‐board processing methods used to implement the BSM and delineate individual dives, models were first fitted to make comparisons between estimations of depth inflection points (bottom phase only), phase duration and vertical speed (for descent and ascent phases only—see Supplementary material S.6 for details of how these were calculated). Models were then fitted for time spent in PrCA behaviours, swimming effort and pitch (for descent and ascent phases only). To aid comparability between model parameters, all data were standardised prior to model input (subtract mean and divide by standard deviation of combined archival and transmitted estimates to retain 1:1 expectation). Across all analyses, a random effect (intercept and/or slope) of individual was included if this lowered the Akaike information criterion (Zuur, Ieno, Walker, Saveliev, & Smith, 2009). Pseudo *r*‐squared (*R* ^2^) values were used to assess correlations between abstracted and archival data, and were calculated according to Nakagawa and Schielzeth (2013) and Johnson (2014), using the muMin package (Barton, 2015) in r. Marginal *R* ^2^ values correspond to only the fixed component of a model whilst conditional *R* ^2^ values correspond to the entire model (composed of both fixed and random effects). In addition, the root mean square error (*RMSE*) was calculated for the fitted values of the model vs. the retrieved archival outputs, alongside the transmitted estimates vs. retrieved archival outputs (using both standardised and raw datasets). Finally, because archival datasets included a record of second resolution detections of PrCA behaviours from the on‐board processing algorithms, confusion matrices were constructed (using the SDMtools package in r; Van der Wal, Falconi, Januchowski, Shoo, & Storlie, 2014) and used to assess how well these classifications reflected those from established archival methods (this analysis included all retrieved archival data, including dives that were not transmitted).

## 3 RESULTS

### 3.1 Overview of transmitted data

**Figure 3:**
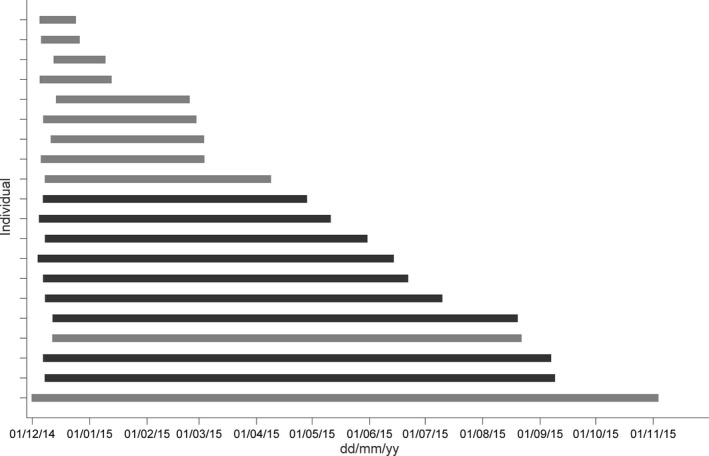
Light (tag not retrieved) and dark (tag retrieved) grey bars mark the period from when the first dive was recorded by each tag to the end of the last dive recorded

**Figure 4:**
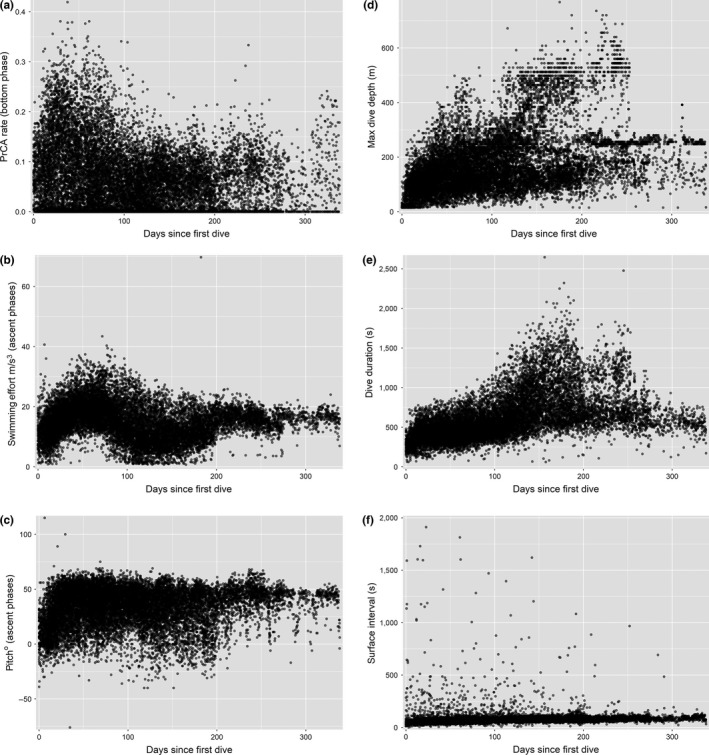
Example data transmitted by DSA tags across deployments lasting upwards of 300 days. Data is pooled from all individuals for: (a) prey catch attempt (PrCA) behaviour rates during bottom phases (time spent in PrCA behaviours scaled by duration), (b) per second average swimming efforts during ascent phases, (c) average pitches during ascent phases, (d) maximum dive depths, (e) dive durations and (f) surface intervals

Useable data were transmitted from all 20 equipped individuals for a total (after data cleaning) of 16,034 dives across 3,023.4 days of sampling (Figures 1 and 3; individual specifics in Supplementary material S.1). The number of dives transmitted per individual ranged from 74 to 1,895, and averaged 5.58 ± 2.35 (±*SD*) dives per day. Sampling periods per individual (time from the first transmitted dive to the end of the last dive) ranged from 19.6 to 338.1 days (Figure 3). 42.2% (6,772) of transmitted dives and 42.6% (1,288.46) of sampling days came from 11 individuals from which tags were not retrieved (Figures 1 and 3). Selected plots of obtained information are shown in Figure 4.

### 3.2 Comparison to retrieved archival data

For the nine individuals from whom tags were retrieved, only 47.1% of sampled dives were transmitted (a total of 10,562 out of 22,420 before cleaning).

#### 3.2.1 Depths, durations and vertical speeds

**Figure 5:**
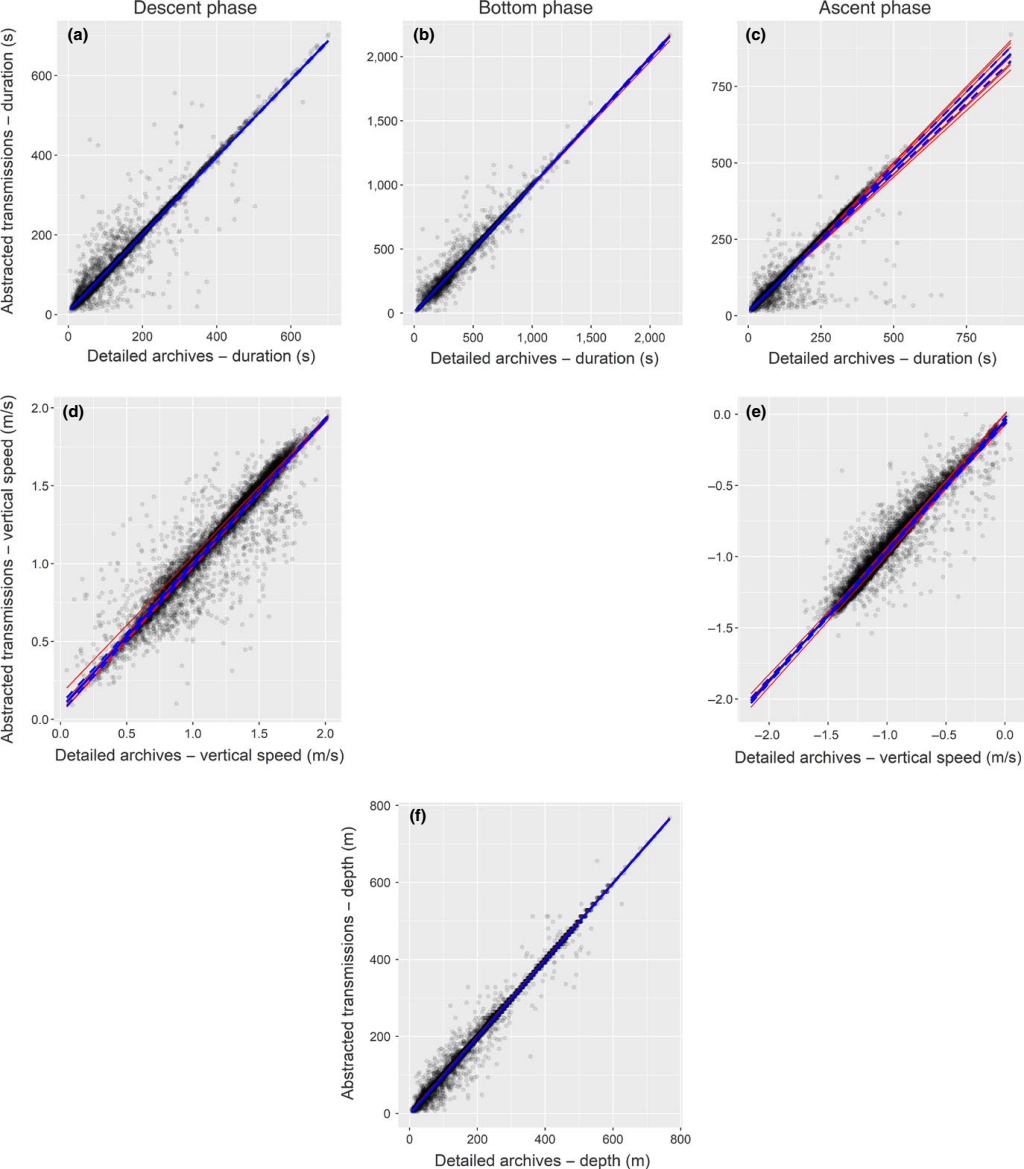
Comparisons between depth‐based outputs from abstracted transmissions and detailed archives. Each red line represents the intercept‐slope output for an individual, the blue line the population mean (95% confidence intervals in dashed blue lines), and black points the raw data. Columns from left to right correspond to descent (a and d), bottom (b and f) and ascent (c and e) phases. Rows, from top to bottom, correspond to phase durations (a:c), vertical speeds (d and e), and inflection point depths (f)

Transmitted depth inflection points, phase durations and vertical speeds showed negligible signs of bias, were strongly correlated with archival estimates (marginal *R* ^2^ values greater than .9) and had small *RMSE*'s (Figure 5 and Table 1). Depth corrections applied to archival datasets varied from −9 m to −0.5 m and averaged −4.4 ± 2.4 m.

**Table 1:** Modelling results from linear mixed effects models used to assess correlations between abstracted (response) and archival (explanatory) estimates for (a) depth associated and (b) accelerometry data. Intercept and slope values close to 0 and 1, respectively, reflect stronger correlations/likeness between the two data types than those far from 0 and 1. Intercept and slope standard deviation values of each model's random component (*R*. intercept and *R*. slope) are reported to evaluate inter‐individual differences in algorithm performances. Pseudo *R* ^2^ values show the amount of variation accounted for by the fixed component of the model (marginal *R* ^2^; $R_{m}^{2}$). and the amount of variation accounted for by random component of the model ($R_{c}^{2}$ (conditional *R* ^2^) − $R_{m}^{2}$). Root mean square errors are shown for the fitted values of the model vs. the retrieved archival data (*RMSE*_mod_), and the transmitted estimates vs. retrieved archival data (*RMSE*_data_; standardised out with brackets and raw within brackets)

	Intercept	Slope	*R*. intercept	*R*. slope	$R_{m}^{2}$	$R_{c}^{2}$ − $R_{m}^{2}$	*RMSE*_mod_	*RMSE*_data_
(a) Depth associated data comparison								
Phase duration								
Descent phase	0.01	0.97	—	—	.949	—	0.03	0.11 (20.84 s)
Bottom phase	0.01	0.99	.01	.01	.978	<.001	0.01	0.17 (31.87 s)
Ascent phase	0.00	0.93	.03	.04	.904	.002	0.05	0.14 (25.86 s)
Vertical speed								
Descent phase	0.06	0.93	.03	.02	.914	.004	0.03	0.08 (0.10 m/s)
Bottom phase	—	—	—	—	—	—	—	—
Ascent phase	−0.03	0.91	.02	.02	.922	.003	0.06	0.09 (0.11 m/s)
Depth								
Descent phase	—	—	—	—	—	—	—	—
Bottom phase	−0.02	1.00	.01	—	.993	<.001	0.02	0.09 (9.69 m)
Ascent phase	—	—	—	—	—	—	—	—
(b) Accelerometry data comparison								
PrCA								
Descent phase	−0.08	0.69	.06	.12	.462	.016	0.10	0.21 (3.19 s)
Bottom phase	0.36	0.69	.11	.05	.650	.012	0.52	0.91 (13.98 s)
Ascent phase	0.00	0.75	.09	.16	.521	.037	0.13	0.23 (3.51 s)
Swimming effort								
Descent phase	3.92	4.13	.33	.33	.858	.016	1.32	1.33 (8.99 m/s^3^)
Bottom phase	5.00	5.04	.76	.79	.734	.038	1.86	1.93 (13.00 m/s^3^)
Ascent phase	3.43	3.57	.38	.47	.846	.035	1.76	1.79 (11.20 m/s^3^)
Pitch								
Descent phase	−0.26	0.72	.09	.10	.599	.020	0.05	0.12 (3.78°)
Bottom phase	—	—	—	—	—	—	—	—
Ascent phase	0.11	0.90	.03	.03	.886	.003	0.04	0.11 (2.83°)

#### 3.2.2 PrCA behaviours

**Figure 6:**
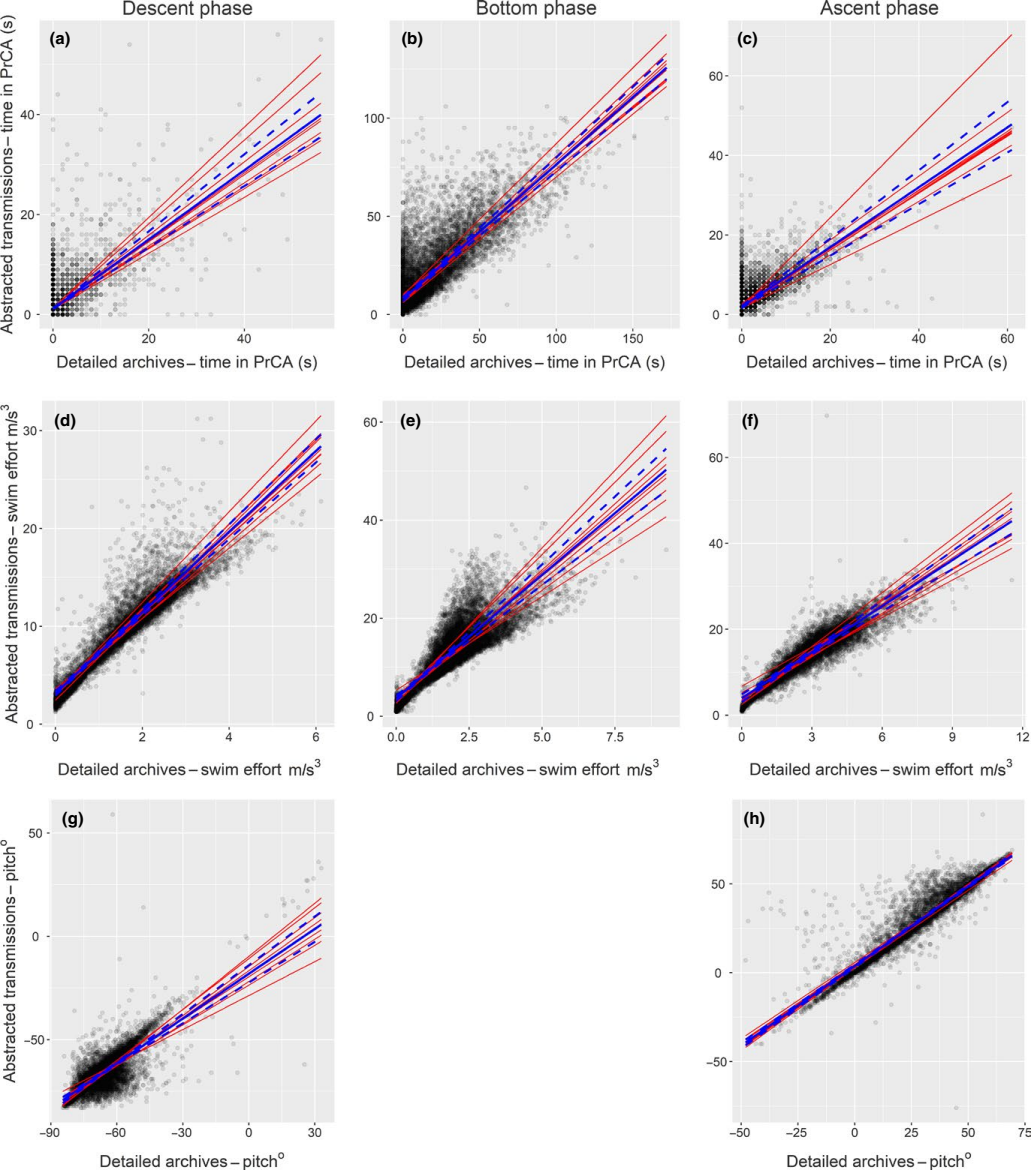
Comparisons between accelerometer based outputs from abstracted transmissions and detailed archives. Each red line represents the intercept‐slope output for an individual, the blue line the population mean (95% confidence intervals in dashed blue lines), and black points the raw data. Columns, from left to right, correspond to descent (a, d and g), bottom (b & e) and ascent (c, f and h) phases. Rows, from top to bottom, correspond to time in prey capture attempt (PrCA) behaviours (a:c), swimming effort (d:f) and pitch (g and h)

Transmitted times spent in PrCA behaviours were positively biased to those from archival processing during bottom phases (when *RMSE*'s were also highest; Figure 6 and Table 1). Bias during descent and ascent phases was negligible. However, marginal *R* ^2^ values of .65 during bottom phases were at least 28.6% higher than those observed for descent and ascent phases (which were 0.46 and 0.52 respectively). Performance metrics from confusion matrices varied with dive phase, with no one phase consistently displaying favourable values (Table 2).

**Table 2:** Performance metrics from confusion matrices of predicted (transmitted) and actual (archival) PrCA behaviour detections. Values below show the mean of all individuals ± the standard deviation, with the best performing dive phase(s) highlighted in bold

Performance metric	Description	Decent phase	Bottom phase	Ascent phase
True positive rate	True positive/actual positive	0.569 ± 0.022	0.574 ± 0.025	**0.632 ± 0.026**
False positive rate	False positive/actual negative	**0.013 ± 0.001**	0.031 ± 0.003	**0.013 ± 0.004**
Specificity	True negative/actual negative	**0.987 ± 0.001**	0.969 ± 0.003	**0.987 ± 0.004**
Precision	True positive/predicted positive	0.255 ± 0.061	**0.489 ± 0.063**	0.391 ± 0.108
Misclassification	(False positive + false negative)/total	**0.016 ± 0.002**	0.051 ± 0.008	0.018 ± 0.004
Accuracy	(True positive + true negative)/total	**0.984 ± 0.002**	0.949 ± 0.008	0.982 ± 0.004
Prevalence	Actual positive/total	0.008 ± 0.003	**0.050 ± 0.013**	0.013 ± 0.003

#### 3.2.3 Swimming effort

Transmitted swimming efforts were positively biased compared to archival estimates (Figure 6 and Table 1). The influence of this bias was more pronounced during the bottom phase of a dive. Transmitted and archival swimming efforts were more strongly correlated during descent and ascent phases, and had marginal *R* ^2^ values which were at least 15.3% higher than those of bottom phases (.86 and .85 vs. .73 respectively). This reflected lower levels of variation in the data (Figure 6e). *RMSE*s were highest during the bottom phase of a dive (Table 1).

#### 3.2.4 Pitch

Compared to archival estimates, transmitted pitches were negatively biased for descent phases and positively biased during ascent phases, due to a systematic over estimation in the angles of descent and ascent (Figure 6 and Table 1). This bias was more pronounced during descent phases. Correlations between transmitted and archival pitches were weaker during descent phases, which had marginal *R* ^2^ values that were 32.4% less than those for ascent phases (.60 vs. .89 respectively). *RMSE*'s were highest during descent phases.

#### 3.2.5 Algorithm performance across individuals

Standard deviations of individual parameter estimates from the random component of models comparing transmitted and archival estimates of depth, phase duration, vertical speed, PrCA behaviours, swimming effort, and pitch, alongside small conditional *R* ^2^ values (all less than .04—representing an increase of, at most, 7.1% from marginal *R* ^2^ values) reflect little variation between individuals in biases from transmitted estimates and correlations with archival data (Table 1, Figures 5 and 6).

## 4 DISCUSSION

In this study, we present a new method for remotely obtaining quantifiable measurements of marine predator behaviours via the combined use of accelerometers, time‐depth recorders and the Argos satellite system. Using juvenile southern elephant seals as a case study, we show that through the use of an intermittent sampling regime, detailed snapshots of an individual's behaviour can be collected over deployment periods exceeding several months to almost a year. Although only 45% of these dives were successfully transmitted (likely due to surfacing times and satellite availability), information was received from over five dives per individual per day. Dependent on dive phase, corresponding estimates of depth based metrics, PrCA behaviours, swimming effort, and descent and ascent pitches were generally comparable to those obtained via the detailed analysis of retrieved archival data, although for some parameters improvements could be made.

### 4.1 Depths, durations and vertical speeds

Transmitted estimates of phase durations, vertical speeds and depth inflection points showed negligible signs of bias and were strongly correlated to corresponding archival outputs, thus validating the performance of the on‐board BSM, alongside the use of the wet‐dry sensor to delineate the start and end of dives. Moreover, these results suggest the effect of sensor drift was small, which is reiterated by the small size of the zero offset depth correction applied to archival datasets (averaging <3% of average inflection point depths, and never more than 6%—9 m).

### 4.2 PrCA behaviours

Estimates of PrCA behaviours from transmitted data included a number of false detections and misclassifications (Table 2), and were positively biased during bottom phases. This likely stemmed from the misidentification of erratic movements and/or signal noise as PrCA behaviours, possibly because the 5 m/s^2^ detection threshold used in on‐board processing algorithms was too low. To obtain similar algorithm performance to that from adult datasets, a threshold of 7 m/s^2^ would be required (see Supplementary materials S.7 vs. S.2). Further improvements could also be made by increasing the length of the time window over which var*A* is calculated (see Section 2), which increased true positive rates (Supplementary material S.8) and possibly reflects the sensitivity of the current algorithm to small spikes in acceleration during periods of relative calm. Nonetheless, future studies may benefit from the use of a more flexible approach that negates the need for prior knowledge of PrCA behaviours in algorithm development, and allows for potential changes in the magnitudes of PrCA movements as individuals develop their foraging abilities and grow. However, potential alternatives need to work on a dive by dive basis (so cannot incorporate two‐state clustering methods), and have minimal computing power requirements. One such solution could be to use the distributions of acceleration standard deviations/variances to identify outlying extreme events (e.g. those out with the limit of a normal distribution or above a multiple number of standard deviations above the mean). Whilst developing such improvements would benefit from more in‐depth validation via the use of animal attached videography (Volpov, Hoskins, et al., 2015; Watanabe & Takahashi, 2013) or further accelerometer measurements (e.g. from jaw attachments; Viviant et al., 2010), the techniques applied to archival data to provide a baseline validation reference in this study have been demonstrated sufficient for the identification of PrCA behaviours across a number of studies (Carroll et al., 2014; Viviant et al., 2010; Volpov, Hoskins, et al., 2015; Ydesen et al., 2014).

Correlations between transmitted and archival estimates of time spent PrCA behaviours during bottom phases were good (~0.65), and variation in algorithm performance between individuals minimal (Table 1). This is in contrast with comparative outputs from descent and ascent phases (with marginal *R* ^2^ values <.55). However, transmitted times spent in PrCA behaviours during bottom phases were positively biased and had increased *RMSE*'s, whilst confusion matrix performance metrics displayed mixed results. An increase in the presence of foraging behaviours other than prey capture (e.g. prey chase and handling alongside searching; Heerah et al., 2014; Volpov, Hoskins, et al., 2015) during the bottom phase of a dive may drive false detection rates and misclassifications in a manner that correlates well with true detections. Whilst false detections and misclassifications were decreased across descent and ascent phases, simultaneous decreases in the strength of correlations between transmitted and archival phase tallies suggests that these detections occur more randomly, and so should be treated with caution and possibly excluded from further analyses. This should not substantially influence inferences made, since the contribution of information from descent and ascent phases on overall foraging effort estimates is low (across archival data 87% of all PrCA behaviours occurred during bottom phases, which is comparable to that reported in other studies of pinnipeds; Gallon et al., 2013; Heerah et al., 2014; Viviant, Jeanniard‐du‐Dot, Monestiez, Authier, & Guinet, 2016). While on‐board detections of PrCA behaviours showed some inconsistencies to those from archival techniques, the strength and consistency (across individuals) of correlations between outputs from the two at the dive phase scale suggest these data can be tentatively used to examine how foraging effort varies across the tagged individuals.

### 4.3 Swimming effort

Across all dive phases, transmitted swimming efforts were positively biased to those from archival data in a manner that increased with the swimming effort measurement itself (i.e. small transmitted swimming efforts were less positively biased than large values). This overestimation likely reflected the summing of all stroke associated (*y*‐axis) accelerations in on‐board processing, rather than the isolation and averaging of individual amplitudes and rates (as in archival processing). Moreover, during bottom phases, the variance of positive bias in transmitted estimates increased with swimming effort (a trend that was consistent across all individuals; Supplementary material S.9), suggesting other movements (e.g. rolling, turning and rapid head jerks) were included in calculations and not sufficiently removed by the filtering process applied during on‐board processing. Indeed, whilst descent and ascent phases typically involve directed swimming behaviours to and from prey patches at depth, concentrated foraging activity during the bottom phase of a dive increases the likelihood of the inclusion of these behaviours in swimming effort calculations (Gallon et al., 2013; Heerah et al., 2014; Viviant et al., 2016). This may be because the bottom limit of the band pass filter was too low and there was no upper limit. Indeed, addressing these points improved the strength of correlations with archival estimates (Supplementary material S.10). Nonetheless, the consistency (across individuals) and strength of correlations between transmitted and archival estimates from descent and ascent phases suggests these swimming efforts can be used with confidence to, for example, make inferences about changes in body condition (e.g. using the methods described in Biuw, McConnell, Bradshaw, & Fedak, 2003; Richard et al., 2014). Swimming efforts from bottom phases should, however, be treated with caution.

### 4.4 Pitch

Bias in transmitted pitches was stronger during descent than ascent phases, which reflects differences in the proportions of these phases composed of more than one segment (25.5% vs. 13.7%, respectively, after dives with shallow first or last segments were removed; see Supplementary materials S.6 & S.11). In both instances, an overestimation in the angle of descent and/or ascent suggests that, where these phases are composed of more than one segment, the segment not incorporated into on‐board processing algorithms is less steep than the first and last segments used (e.g. the ascent phase in Figure 2). Nonetheless, these differences are generally small and consistent across all measurements (and individuals; Table 1). Whilst the first and last segment pitches of a dive can be treated tentatively as representative of descent and ascent pitches respectively, dives where these phases are composed of more than one segment could be excluded from subsequent analyses to increase reliability (e.g. in the calculation of descent swimming speeds to infer body condition; Biuw et al., 2003; Richard et al., 2014).

## 5 CONCLUSIONS

In this study, we have presented a new approach for remotely obtaining detailed information from accelerometers on the sub‐surface behaviour of a free‐ranging marine predator in its natural environment. While outputs from these methods were comparable to those requiring device retrieval, improvements could be made, for which our study provides valuable insight. The data acquired represent some of the first quantifications of the fine‐scale movements of juvenile southern elephant seals during their first at‐sea foraging trip immediately following weaning. Consistencies in algorithm performance across individuals suggest these outputs can be used to address a number of exciting and novel questions regarding ontogenetic behavioural changes and suspected survival rates. In particular, changes in descent swimming speeds and ascent swimming efforts (Biuw et al., 2003; Richard et al., 2014) can be used as a proxy for body condition, and compared to indices of foraging effort (i.e. time spent in PrCA behaviours) to assess foraging performance (Richard, Cox, Picard, Vacquie‐Garcia, & Guinet, 2016). The methods explored in this study are applicable to a diversity of other species including pinnipeds, cetaceans, seabirds (e.g. penguins) and possibly large fish (for which it is almost impossible to recapture individuals to recover high resolution archival data), and can be used to quantify previously unobservable behaviours and movements across entire geographical ranges. This includes individuals from a variety of age classes and life history stages, alongside those that die whilst at sea and would otherwise be impossible to observe. Subsequent gains in knowledge will significantly contribute to our understanding of the at‐sea ecology of free‐ranging marine predators alongside how marine ecosystems function.

## AUTHORS’ CONTRIBUTIONS

C.G. and I.H. were involved in the SCOUT‐DSA‐296 tag algorithm design. All authors were involved in the conception of ideas and methodological design. F.O., H.W. and C.G. collected the data. S.L.C., M.G. and C.R. analysed the data. S.L.C. led the writing of the manuscript. All authors contributed critically to the drafts and gave final approval for publication.

## DATA ACCESSIBILITY

Matlab and R codes and scripts used to process the transmitted and archival datasets are available at github accounts “SamLCox” and “SESman” under https://doi.org/10.5281/zenodo.809385, https://doi.org/10.5281/zenodo.809383, https://doi.org/10.5281/zenodo.809387 and https://doi.org/10.5281/zenodo.809182. Data used in the comparative analyses of the abstracted transmissions and retrieved archives is available under https://doi.org/10.5061/dryad.vv107 (Cox et al., 2017).
