# Supplementary material for: Processing of acceleration and dive data on‐board satellite relay tags to investigate diving and foraging behaviour in free‐ranging marine predators
Source: Methods Ecol Evol. 2017 Jul 27;9(1):64–77. doi: 10.1111/2041-210X.12845 (PMC5812097; doi:10.1111/2041-210X.12845)
Supplement: Supplementary file 1 [file MEE3-9-64-s001.pdf]

SUPPLEMENTARY MATERIALS

Contents

S.1 Individual deployment metrics 2

S.2 Initial pre-deployment threshold selection for the on-board identification of prey catch attempt (PrCA) behaviours 3

S.3 Power spectral density curves estimated from retrieved archival data 5

S.4 Absolute amplitudes/intensities of flipper stroke accelerations along the lateral (y) axis 6

S.5 Dive phase assignment methods 7

S.6 Preparation of transmitted and archival data for comparative analyses 10

S.6.1 *Data cleaning* 10

S.6.2 *Match-up of archival and transmitted dives* 10

S.6.3 *Data preparation for comparative analyses* 11

S.7 Improved threshold selection, based upon retrieved archival data from juveniles, for the on-board identification of prey catch attempt (PrCA) behaviours 12

S.8 The influence of the size of the averaging time window on the performance of the on-board processing algorithm for identifying prey catch attempt (PrCA) behaviours by juvenile southern elephant seals 15

S.9 Bottom phase correlations for each individual between swimming efforts from archival and transmitted data 17

S.10 Algorithm improvements for the on-board estimation of swimming effort based upon retrieved archival data from juveniles 18

S.11 Comparisons between pitches from transmitted and archival data excluding dives with ascent and descent phases comprising more than one segment 20

S.12 References 21

S.1 Individual deployment metrics

Table S1. Individual southern elephant seal pup IDs alongside DSA tag deployment dates, biometrics taken during deployments, dive recording start/end periods alongside lengths and the total number of dives sampled per individual (after data cleaning but including dives with shallow first and last segments). All dates are shown in a dd/mm/yyyy format.

Pup ID	Sex (F/M)	Tag retrieved	Deployment date	Deployment weight (kg)	Deployment length (cm)	Date of first dive	Date of last dive	DSA dive recording length (days)	Total number of dives
140059	F	Yes	03/12/2014	88	142	11/12/2014	19/08/2015	250.9	1379
140060	M	Yes	03/12/2014	85	140	03/12/2014	14/06/2015	192.1	1201
140061	M	No	03/12/2014	97	147	13/12/2014	23/02/2015	72.1	399
140062	F	Yes	04/12/2014	61.6	130	07/12/2014	08/09/2015	275.3	913
140063	F	Yes	04/12/2014	78	142	04/12/2014	10/05/2015	157.3	808
140064	M	No	04/12/2014	73.4	140	04/12/2014	12/01/2015	38.8	165
140065	M	No	04/12/2014	83.4	141	04/12/2014	24/12/2014	19.6	105
140066	M	No	26/11/2014	64	139	30/11/2014	03/11/2015	338.1	1895
140067	F	No	05/12/2014	60.6	133	05/12/2014	03/03/2015	88.2	539
140068	F	Yes	05/12/2014	88.6	148	06/12/2014	21/06/2015	196.9	1211
140069	F	Yes	05/12/2014	64.8	122	07/12/2014	10/07/2015	214.3	1120
140070	M	No	05/12/2014	98.4	150	05/12/2014	26/12/2014	20.8	74
140071	M	No	05/12/2014	92.4	148	07/12/2014	08/04/2015	122.1	708
140072	M	Yes	06/12/2014	78.8	147	06/12/2014	06/09/2015	274.1	1662
140073	F	Yes	05/12/2014	74.2	132	07/12/2014	30/05/2015	173.9	968
140074	M	No	06/12/2014	47.2	112	12/12/2014	09/01/2015	27.9	109
140075	F	Yes	06/12/2014	59.4	123	06/12/2014	28/04/2015	142.4	979
140076	M	No	06/12/2014	116.4	151	10/12/2014	03/03/2015	82.7	391
140077	F	No	06/12/2014	109.4	149	11/12/2014	21/08/2015	253.1	1030
140078	F	No	06/12/2014	76.6	145	06/12/2014	27/02/2015	82.7	378

S.2 Initial pre-deployment threshold selection for the on-board identification of prey catch attempt (PrCA) behaviours

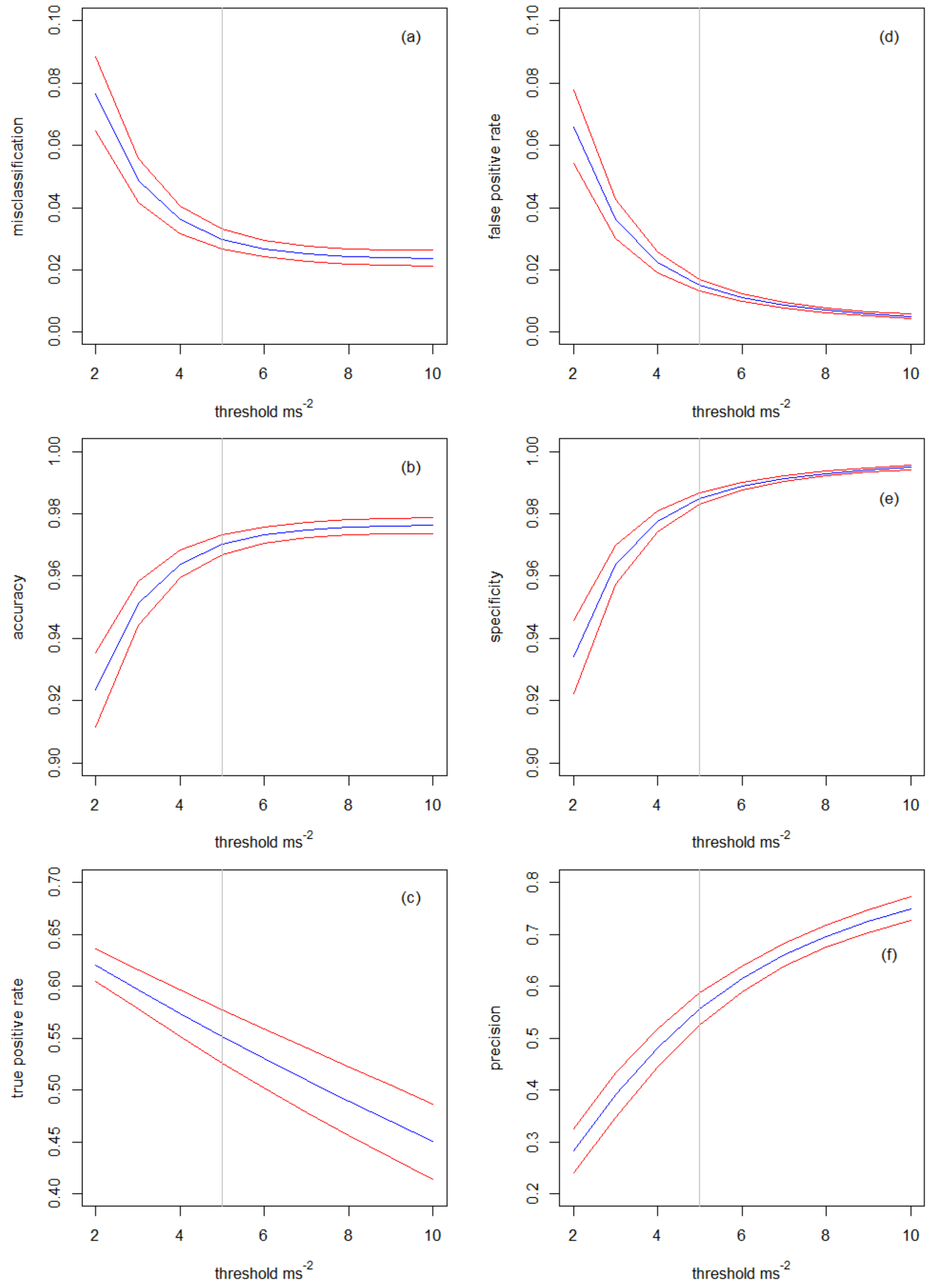

Figure S1. Plotted outputs from confusion matrices used to determine the acceleration threshold used during on-board identification of prey catch attempt (PrCA) behaviours. Due to a lack of available juvenile southern elephant seal data prior to device deployments, threshold selection was preformed using data from 10 randomly selected post-reproduction female southern elephant seal adults from whom archival accelerometer data was available (from deployments occurring between 2013 and 2015). From the top left: (a) misclassification rates at varying thresholds, (b) accuracy at varying thresholds, (c) true positive rates at varying thresholds, (d) false positive rates at varying thresholds, (e) specificity at varying thresholds and (f) precision at varying thresholds. Blue lines show the mean value across all 10 individuals and red lines the standard errors. The vertical grey line in each plot represents the chosen threshold of $5ms^{-2}$.

The pre-transmission identification of prey catch attempt (PrCA) behaviours involved the identification of rapid body and head movements, which have been found to be good indicators of PrCA behaviours across a range species (Viviant *et al.* 2010; Carroll *et al.* 2014; Ydesen *et al.* 2014; Volpov *et al.* 2015). Typically, procedures involve the identification of peaks in variance along each of the acceleration axes (x, y and z) using various types of clustering algorithms (e.g. k-means clustering or Gaussian mixture models; Viviant *et al.* 2010; Chimienti *et al.* 2016). However, such methods are costly in computer processing power and so unsuitable for on-board application. Pre-selected thresholds can be used instead to address this problem, although ideally these should be informed via the use of prior information. For this study, because we did not have access to juvenile southern elephant seal data, we used accelerometer data (from between 2013 and 2015) collected from 10 post-reproduction adult female southern elephant seals to select appropriate thresholds. PrCA behaviours were then identified using pre-transmission on-board processing techniques (see section "*Detection of prey catch attempt (PrCA) behaviours*" of main article) incorporating a range of thresholds ranging from 2 to 10 ms^{-2} in increments of $1ms^{--2}$, and compared to those identified using established clustering algorithms which were assumed to represent 'true' behaviour (e.g. those used in Viviant *et al.* 2010, 2016; Guinet *et al.* 2014; Jouma'a *et al.* 2016; see also section "*Detection of prey catch attempt (PrCA) behaviours*" of main article). To assess the performance of each tested threshold, we used outputs from confusion matrices (from the R package SDMTools; Van der Wal *et al.* 2014) to calculate a range of indicative metrics based upon the true positive/negative and false positive/negative detection rates of the on-board processing algorithm in comparison to the established clustering techniques. Confusion matrices were generated separately for each individual at each threshold. An average of each subsequent metric was then taken across all the individuals at each threshold. Metrics calculated from the confusion matrix outputs include: (1) accuracy – the number of true PrCA behaviours and true non-PrCA behaviours identified by the on-board processing algorithm divided by the total number of observations, (2) misclassification – the number of false PrCA behaviours and false non-PrCA behaviours divided by the total number of observations, (3) the true positive rate – taken as the number of true PrCA behaviours predicted by the on-board processing algorithm divided by the total number of PrCA behaviours identified using the established clustering technique, (4) the false positive rate – the number of false PrCA behaviours identified by the on-board processing algorithm divided by the total number of non-PrCA behaviours identified using the established clustering technique, (5) precision – the number of true PrCA behaviours divided by the total number of predicted (true and false) PrCA behaviours, and (6) specificity – the number of true non-PrCA behaviours divided by the actual number of non-PrCA behaviours (as indicated by established processing techniques). The individual means for each tested threshold were then plotted, and break points in these metrics, that would be indicative of an appropriate threshold value, were visually identified (Figure S1). A value of $5ms^{-2}$ was found to be appropriate across the majority of metric plots. An exception to this was the true positive rate, for which decreasingly lower thresholds consistently resulted in higher true positive rates. However, this resulted in a simultaneous increase in false positive rates and decrease in precision (alongside deterioration of other threshold performance metrics). As such, we took a conservative approach in the selection of the $5ms^{-2}$ threshold to ensure the algorithm was not over-estimating PrCA behaviours, even if this was at a cost of missing some.

S.3 Power spectral density curves estimated from retrieved archival data

Power spectral densities were generated separately for each individual. The location and width of the band pass filter was determined using Lavielle segmentation (Lavielle 1999), the bottom and upper limits of which fell within the 0.49Hz to 2.05Hz range for all nine individuals (Figure S2).

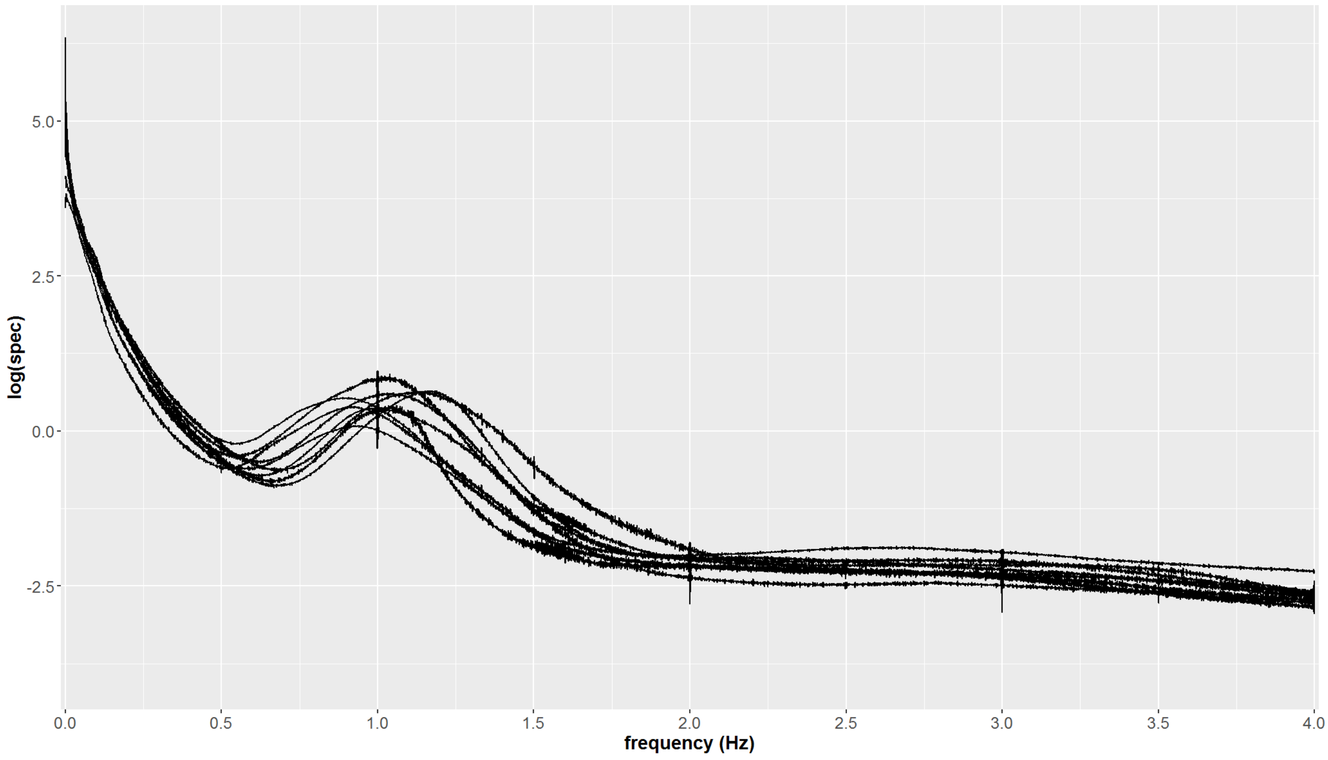

Figure S2. Power spectral density curves of accelerations along the lateral (y) axis. Each line corresponds to one of each of the nine individuals for which archival data was retrieved.

S.4 Absolute amplitudes/intensities of flipper stroke accelerations along the lateral (y) axis

To determine the threshold below which filtered acceleration movements along the lateral (y) axis were not associated with directed swimming movements, histograms of acceleration amplitudes/intensities were constructed by taking the absolute difference between the peaks and troughs of the acceleration signal (Sato *et al.* 2003; Aoki *et al.* 2011). Piecewise regressions with a single break point were then constructed for each individual. The outputs of these are shown in Figure S3. A threshold of $0.2 ms^{-2}$ was found to be an appropriate cut-off value.

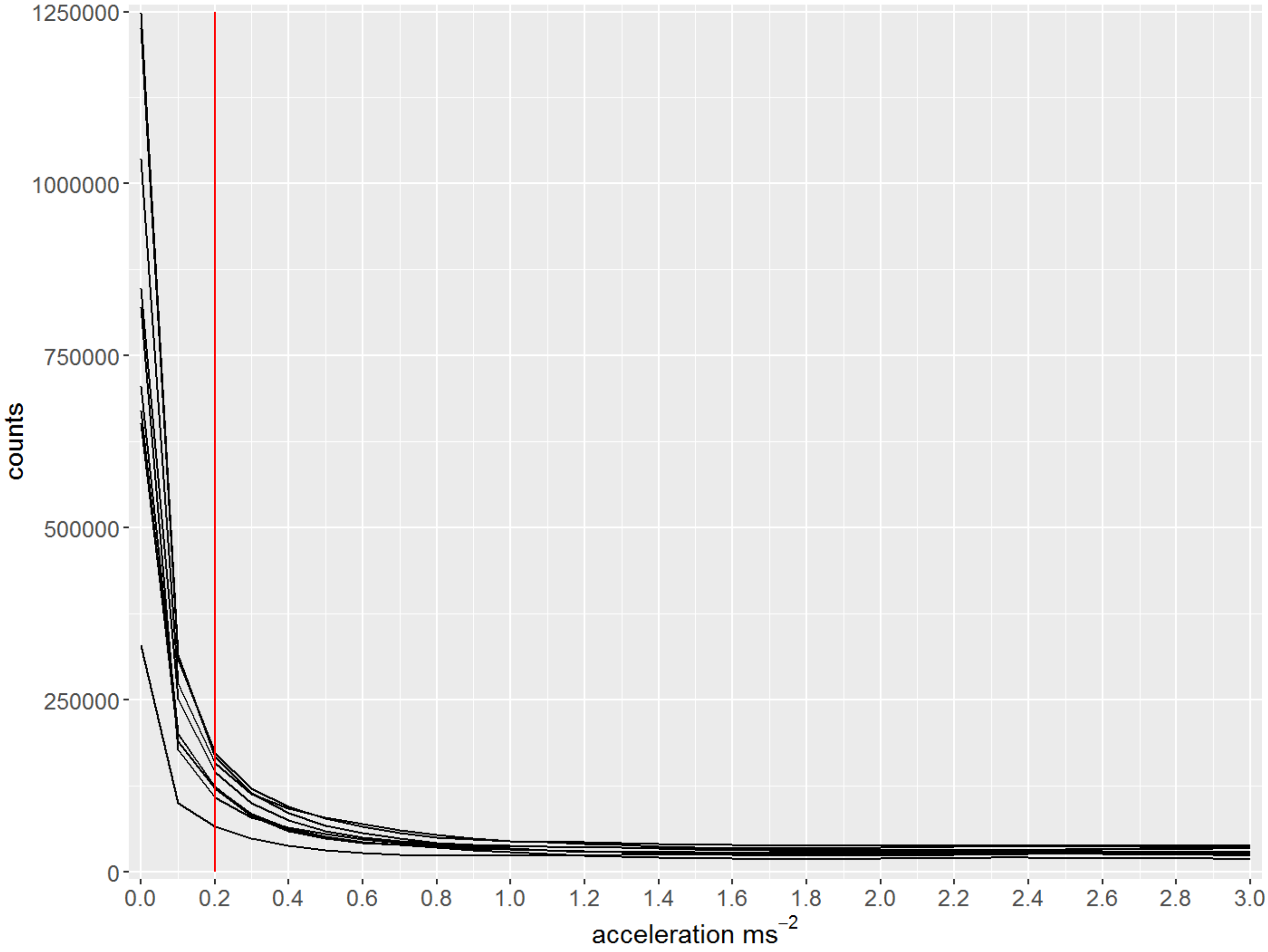

Figure S3. Outputs from piecewise regression models composing a single breakpoint for each of the nine individuals from whom archival data was retrieved. The vertical red line shows the position of the selected threshold ($0.2 ms^{-2}$) used to isolate noise signals from those associated with swim stroke motions.

S.5 Dive phase assignment methods

Segments for each dive from the abstracted and processed detailed data were grouped into descent, bottom and ascent phases. For both datasets (transmitted abstractions and retrieved archival), this was performed post-hoc (i.e. the following methods described did not occur on-board the DSA tag). The first and last segments of a dive were always assigned to descent and ascent phases respectively. Out with this, the bottom phase ran from the first segment at which the vertical speed dropped below $0.42ms^{-1}$ to the first segment where the vertical speed dropped below $-0.76ms^{-1}$. To determine the values of these thresholds, the bottom phases of a random selection of 20 dives per individual (totalling 360 across the nine pups) were manually allocated and compared to allocations using a selection of test threshold values from an automated algorithm (that identified the first and last segments in a dive profile where the vertical speed fell below set threshold values). The range of test threshold values was based upon visual observations of vertical speeds against manually assigned dive phases. For the start of the bottom phase these spanned 0.3 to $0.6ms^{-1}$ at intervals of 0.01, and for the end of the bottom phase from -0.9 to $-0.6ms^{-1}$ (also at intervals of 0.01). The most appropriate thresholds were chosen as those that (1) maximized the percentage of perfect matches to the manually assigned phases and (2) minimized the absolute difference between the segments marking the beginning and end of the bottom phase (Figure S4). During bottom phases, individuals sometimes made erratic movements (Figure S5), which falsely triggered the end of a bottom phase. In these instances, the vertical speed of the third segment was replaced with a smoothed value based upon outputs from a loess smoother applied across all five segments. This improved the percentage perfect match rate across all tested threshold values for the end of the bottom phase by ~10%. The other segment vertical speeds were not replaced with smoothed values as this decreased the ability of the algorithm to detect the beginning and end of the bottom phase. The descent phase corresponded to those segments preceding the bottom phase, and the ascent phase to those following the bottom phase. Example allocations of dive bottom phases are shown in Figure S5.

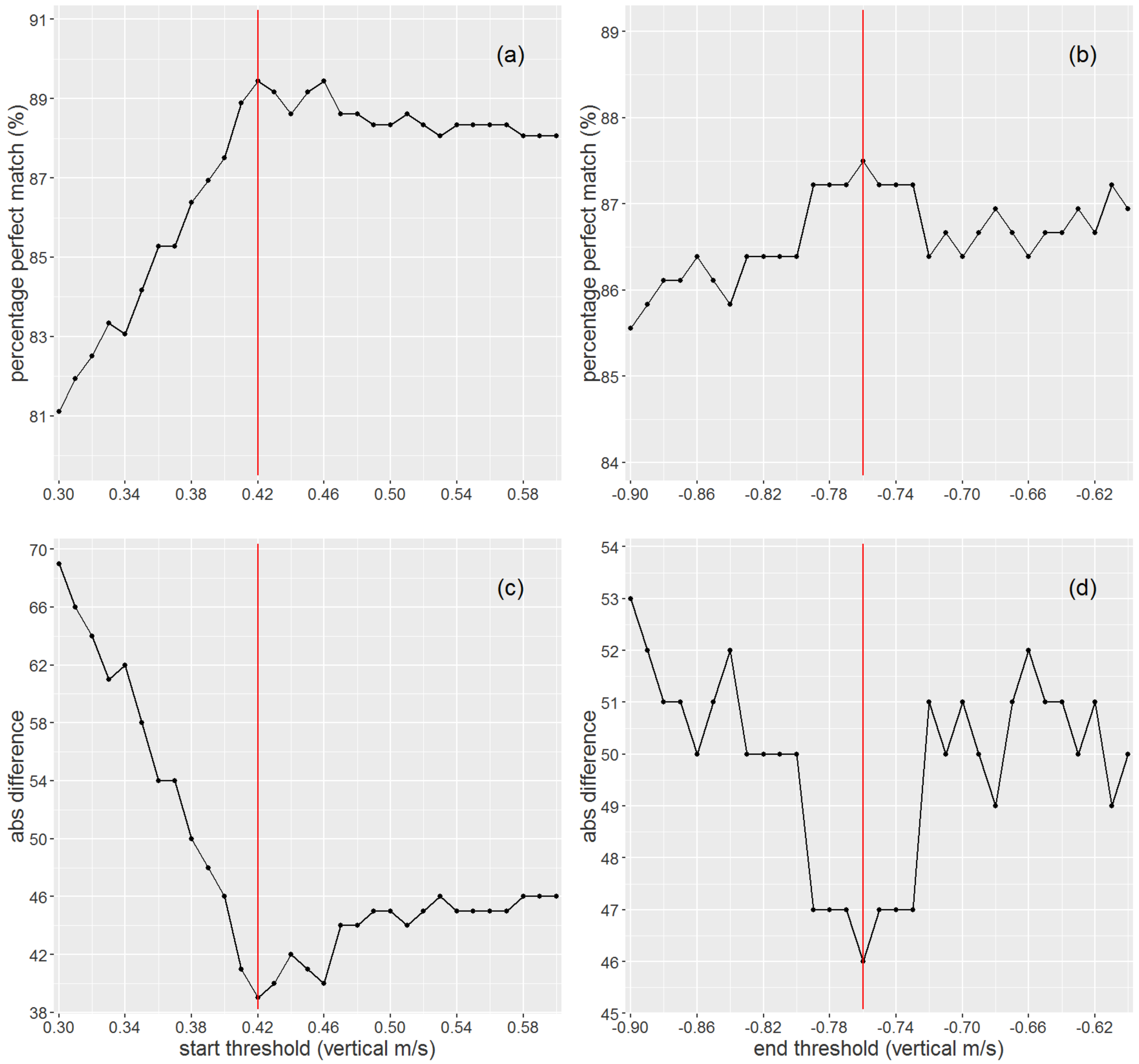

Figure S4. Plots used in the selection of threshold vertical speed values to determine the beginning and end of the bottom phase of a dive. From the top left: (a) the percentage perfect matches between the manually assigned phase beginnings and those from the algorithm using a range of test threshold values, (b) the percentage perfect matches between the manually assigned phase ends and those from the algorithm using a range of test threshold values, (c) the absolute difference in the segment IDs that marked the beginning of the bottom phase via manual assignment and automated techniques over a range of test threshold values, and (d) the absolute difference in the segment IDs that marked the end of the bottom phase via manual assignment and automated techniques over a range of test threshold values.

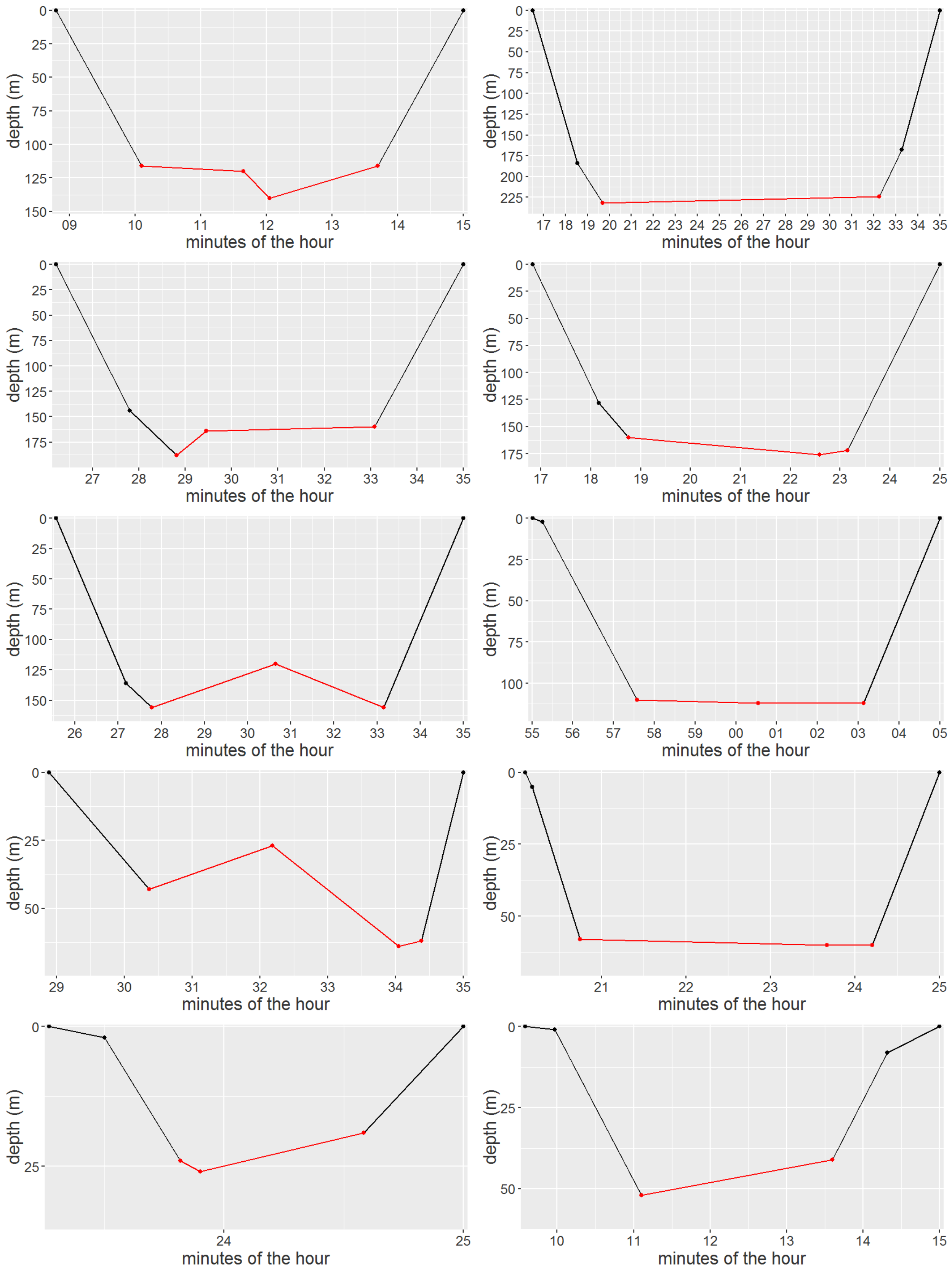

Figure S5. Example dive profiles from six individuals with bottom phases highlighted in red.

S.6 Preparation of transmitted and archival data for comparative analyses

S.6.1 Data cleaning

Prior to comparative analyses, the archival and transmitted data were cleaned to remove erroneous measurements. This included dives with vertical speeds greater than 3.2ms^{-1} (i.e. greater than the 99% vertical speed quantile observed in female adult southern elephant seals; Guinet unpublished data), durations of more than 2800 seconds and depths greater than 1200m (depth and duration thresholds based on the identification of extreme outliers via histograms and dot plots; Zuur *et al.* 2010). For the transmitted data, a number of repeat transmissions alongside dives where the wet-dry sensor had not correctly identified the start and/or end of a dive were also removed (i.e. dives with internal segments with 0m depth; in total these represented only ~3.4% of all sampled dives). Finally, the brokenstick model (BSM) occasionally inserted a breakpoint in very shallow water at the beginning or end or a dive (e.g. bottom two subplots of Figure S5), creating short and shallow segments with misrepresentative descent and ascent pitches and swimming efforts. These were filtered out and excluded from comparative analyses (but included in general information on the number of dives obtained per individual etc). To identify such scenarios, histograms of the differences in vertical speed between the first and second segment and the fourth and fifth segment were constructed (histograms of pitch and vertical speed from segments one and five did not produce clear breakpoints; Figure S6). Dives with differences in vertical speeds of more than 0ms^{-1} for segments two minus one and 0.2ms^{-1} for segments five minus four were excluded from further analyses. This totalled 838 dives (8.26% of data before this filtering step but after other data cleaning procedures).

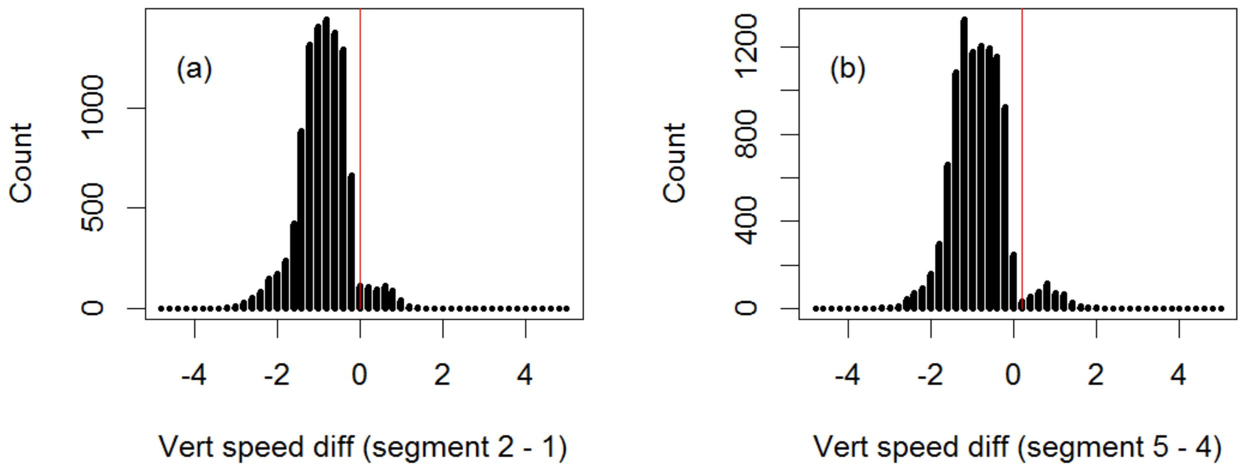

Figure S6. From left to right, histograms of differences in vertical speeds between (a) segments two and one, and (b) segments five and four. Red lines represent thresholds above which a dive was excluded from further analyses (0ms^{-1} for segments two minus one and 0.2ms^{-1} for segments five minus four).

S.6.2 Match-up of archival and transmitted dives

Archived dives were matched to the nearest abstracted dive using the time stamp of the start of a dive (exact time matches were not used due to a slight temporal mismatch between the two data types due to clock drift and synchronisation). To ensure dives were correctly paired between the two datasets, for a

match to be retained three further criteria had to be met: (1) an archived dive must occur within 10 minutes of the transmitted dive it is matched to, (2) the maximum depth of an archived dive must be within 10m of that of the transmitted dive it is matched to, and (3) the total duration of an archived dive must be within two minutes of that of the transmitted dive it is matched to. Given that the sampling protocol of the DSA tag meant that, at a maximum, only one dive was sampled every two hours, these criteria were sufficient to accurately match dives from the two datasets.

S.6.3 Data preparation for comparative analyses

Because behavioural differences between descent, bottom and ascent phases may impact the performance of on-board processing algorithms (and thus quality of transmitted data), comparative analyses between the abstracted and retrieved archival data were conducted separately for each of these phases. For each dive phase, the time spent in PrCA behaviours was summed. The total swimming effort was summed, and divided by the total duration of the dive phase to give a per second average (Jouma'a *et al.* 2016). For abstracted transmissions, the descent pitch corresponded to the first segment of a dive and the ascent pitch to the last segment, even if these phases comprised multiple segments. For the archival data, the pitch of the descent and ascent phases was the average pitch of its comprised segments (weighted by the duration of each segment). This was so we could assess if transmitted pitches from the first and last segments of a dive are representative of those from the entire descent and ascent phases. To validate the on-board processing methods used to implement the BSM and delineate a dive, we also assessed the comparability of a number of depth based metrics. This included the initial and final depths of each phase, alongside its duration. Finally, for abstracted transmissions, descent and ascent vertical speeds were taken as the weighted (by duration) average of the vertical speed calculated from the initial and final depths alongside the duration of each corresponding segment, whilst for the retrieved archival data vertical speed was taken as the average (weighted by the duration of each segment) of previously calculated segment estimates (which incorporated all depth measurements (1Hz), and so reflected small fluctuations in depth within a dive phase).

S.7 Improved threshold selection, based upon retrieved archival data from juveniles, for the on-board identification of prey catch attempt (PrCA) behaviours

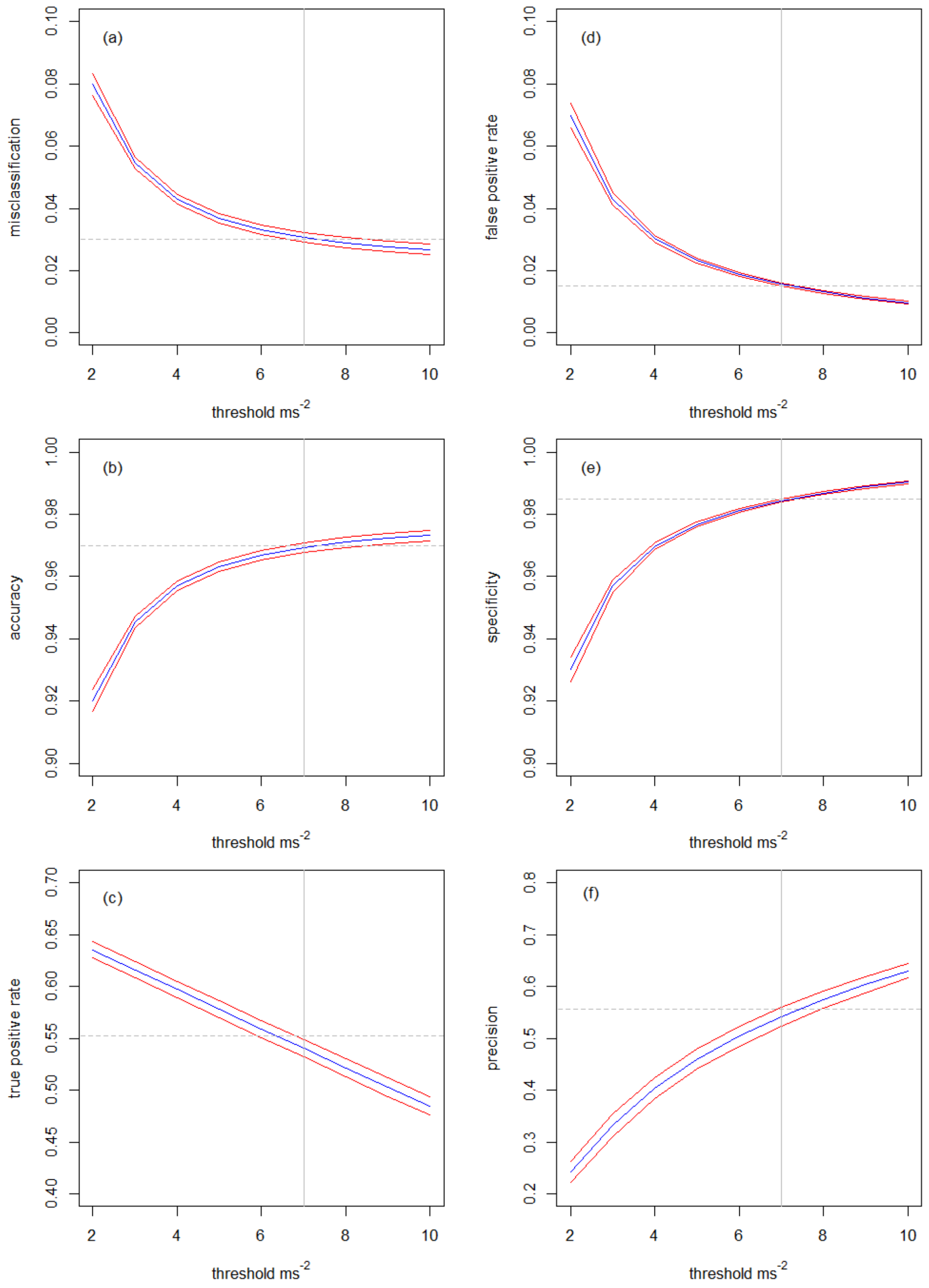

Figure S7. Plotted outputs from confusion matrices used to determine an improved acceleration threshold for on-board identification of prey catch attempt (PrCA) behaviours via the analysis of retrieved archival data. From the top left: (a) misclassification rates at varying thresholds, (b) accuracy at varying thresholds, (c) true positive rates at varying thresholds, (d) false positive rates at varying thresholds, (e) specificity at varying thresholds and (f) precision at varying thresholds. Blue lines show the mean value across all 10 individuals and red lines the standard errors. The horizontal grey dashed line in each plot represents the performance of the chosen threshold of 5ms^{-2} on adult data (see section S.2 of this document). The vertical grey line corresponds to the new threshold values (7ms^{-2}) required to match this performance with the juvenile data.

To assess the performance of a range of new acceleration thresholds for the on-board processing of PrCA behaviours, we used outputs from confusion matrices (from the R package SDMTools; Van der Wal *et al.* 2014) to calculate a range of indicative metrics that reflected true positive/negative and false positive/negative detection rates across the retrieved juvenile southern elephant seal dataset. For this, it was assumed that outputs from established archival processing techniques represented "true" behaviour (see section "*Detection of prey catch attempt (PrCA) behaviours*" of methods of main article). Confusion matrices were generated separately for each individual at each threshold. An average of each subsequent metric was then taken across all the individuals at each threshold. Metrics calculated from the confusion matrix outputs were the same as those described in section S.2 of this document and are plotted in Figure S7 above. A value of $7 ms^{-2}$ was found to produce similar results to those obtained from algorithms developed upon adult female southern elephant seals (see also section S.2 of this document).

Using outputs from data processed using this new threshold, the time spent in PrCA behaviours per dive phase was then calculated and compared to estimates from archival processing (as described in section "*Comparative analyses between transmitted abstracted data and retrieved archival data*" of the methods of the main article). Across all phases, a substantial improvement was seen in correlations between the two data types (see model outputs from Figure S8 and Table S2 below in comparison to Figure 6 and Table 1 of the main article).

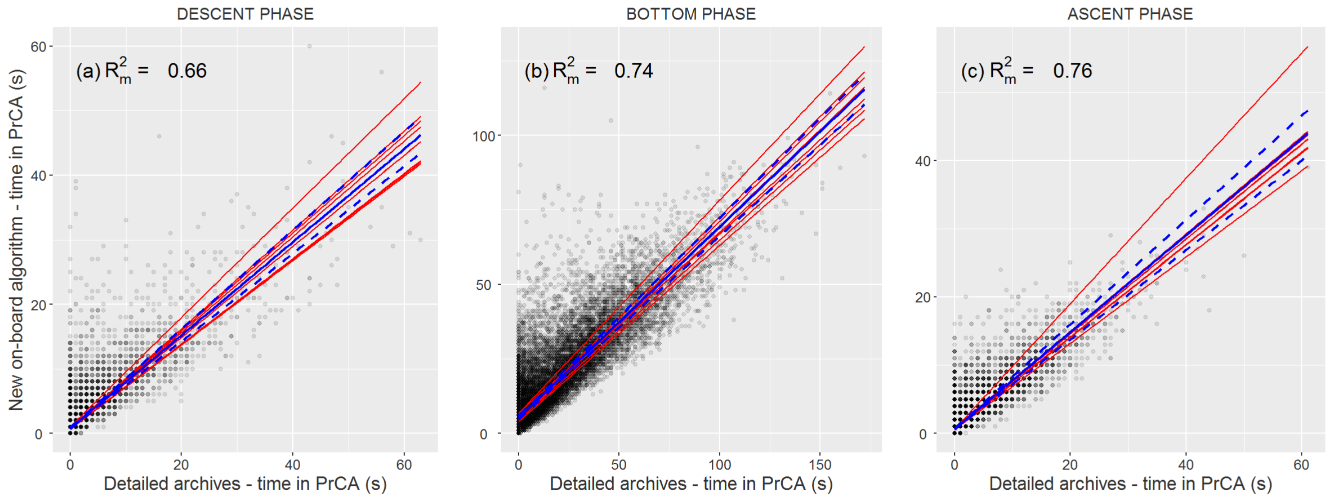

Figure S8. Comparisons between estimates of time in PrCA behaviours from on-board processing techniques using a new acceleration algorithm of $7 ms^{-2}$, and detailed archives. Each red line represents the intercept slope output for each individual, the blue line the population mean (95% confidence intervals in dashed blue lines), and black points the raw data. From left to right: (a) descent phases, (b) bottom phases and (c) ascent phases.

Table S2. Modelling results from linear mixed effects models used to assess correlations between estimates of PrCA behaviours from abstracted (response) data processed using a new threshold of $7ms^{-2}$ and archival (explanatory) estimates. Intercept and slope values close to 0 and 1 respectively reflect stronger correlations/likeness between the two data types than those far from 0 and 1. Intercept and slope standard deviation values of each model's random component (R. intercept and R. slope) are reported to evaluate inter-individual differences in algorithm performances. Pseudo R^2 values show the amount of variation accounted for by the fixed component of the model (marginal R^2; R^2_m) and the amount of variation accounted for by random component of the model (R^2_c (conditional R^2) - R^2_m). Root mean square errors are shown for the fitted values of the model versus the retrieved archival data ($RMSE_{mod}$), and the transmitted estimates versus retrieved archival data ($RMSE_{data}$; standardised out with brackets and raw within brackets).

	Intercept	Slope	R. intercept	R. slope	R^2_m	R^2_c- R^2_m	$RMSE_{mod}$	$RMSE_{data}$
Descent phase	-0.07	0.72	0.04	0.06	0.657	0.008	0.09	0.17 (2.42 s)
Bottom phase	0.21	0.64	0.08	0.04	0.744	0.010	0.59	0.84 (11.71 s)
Ascent phase	-0.09	0.71	0.04	0.08	0.760	0.012	0.09	0.15 (2.01 s)

S.8 The influence of the size of the averaging time window on the performance of the on-board processing algorithm for identifying prey catch attempt (PrCA) behaviours by juvenile southern elephant seals

To assess how changes in the size of time window over which *varA* was calculated in the on-board processing of PrCA behaviours (see section "*Detection of prey catch attempt (PrCA) behaviours"* of methods of main article) may impact algorithm performance, we again used outputs from confusion matrices (from the R package SDMTools; Van der Wal *et al.* 2014) to calculate a range of indicative metrics reflective of true positive/negative and false positive/negative detection rates. For this, it was assumed that outputs from established archival processing techniques represented "true" behaviours (see section "*Detection of prey catch attempt (PrCA) behaviours"* of methods of main article). Confusion matrices were generated separately for each individual at each threshold. An average of each subsequent metric was then taken across all the individuals at each threshold. Metrics calculated from the confusion matrix outputs were the same as those described in section S.2 of this document and are plotted in Figure S9 below. Varying the time window over which *varS* was calculated did not influence the performance of most metrics, except for the true positive rate, which increased with increasing averaging window sizes. As such, in addition to increasing the threshold above which variance in acceleration translates into PrCA behaviours (see section S.7 of this document), future applications of the on-board processing algorithm could also alter the size of this window to further improve the performance of the method.

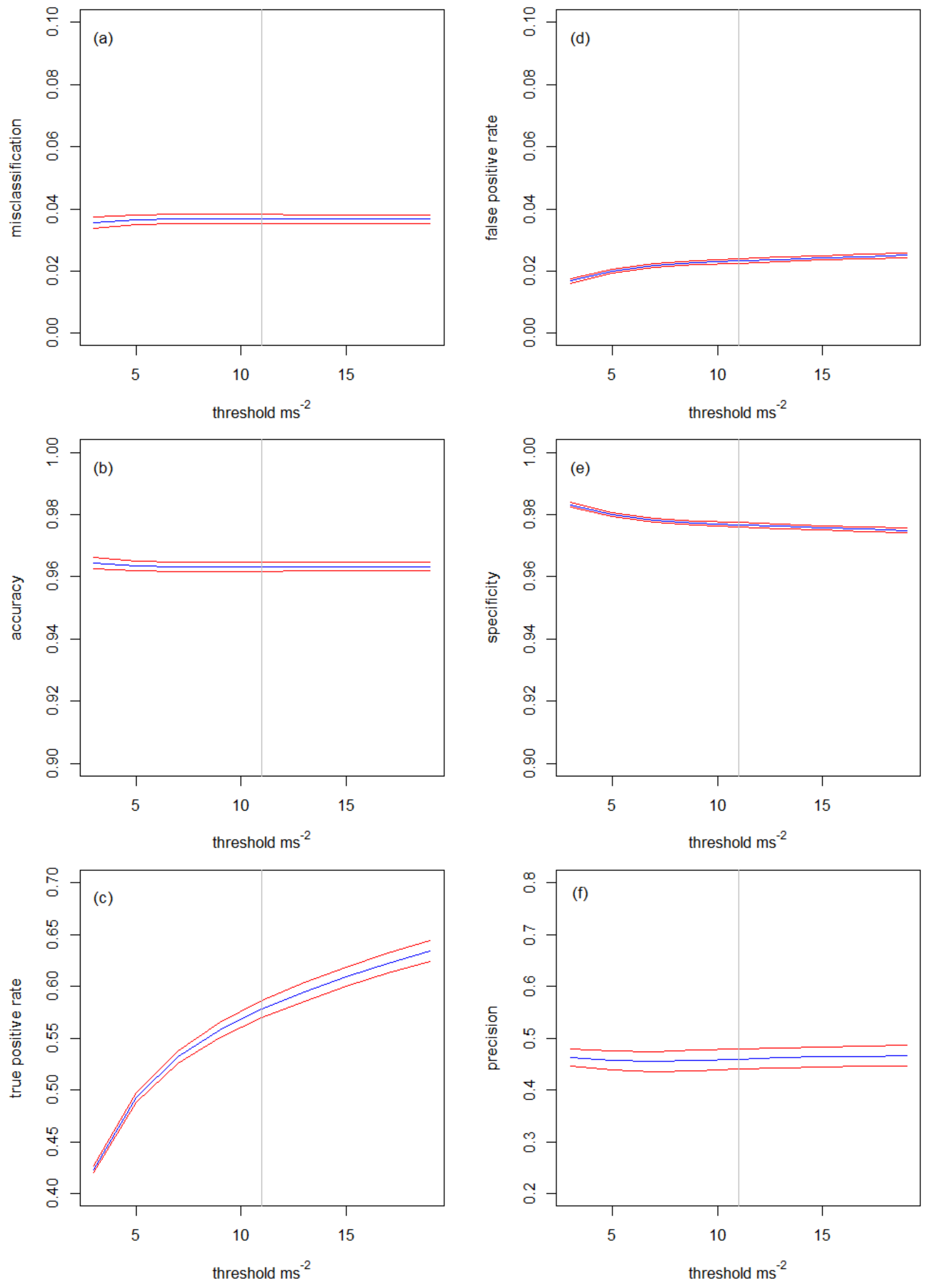

Figure S9. Plotted outputs from confusion matrices used to determine how varying the size of the time averaging windows used during on-board identification of prey catch attempt (PrCA) behaviours influences overall algorithm performance. From the top left: (a) misclassification rates at varying thresholds, (b) accuracy at varying thresholds, (c) true positive rates at varying thresholds, (d) false positive rates at varying thresholds, (e) specificity at varying thresholds and (f) precision at varying thresholds. Blue lines show the mean value across all 10 individuals and red lines the standard errors. The vertical grey line in each plot represents the current value of 11 seconds.

S.9 Bottom phase correlations for each individual between swimming efforts from archival and transmitted data

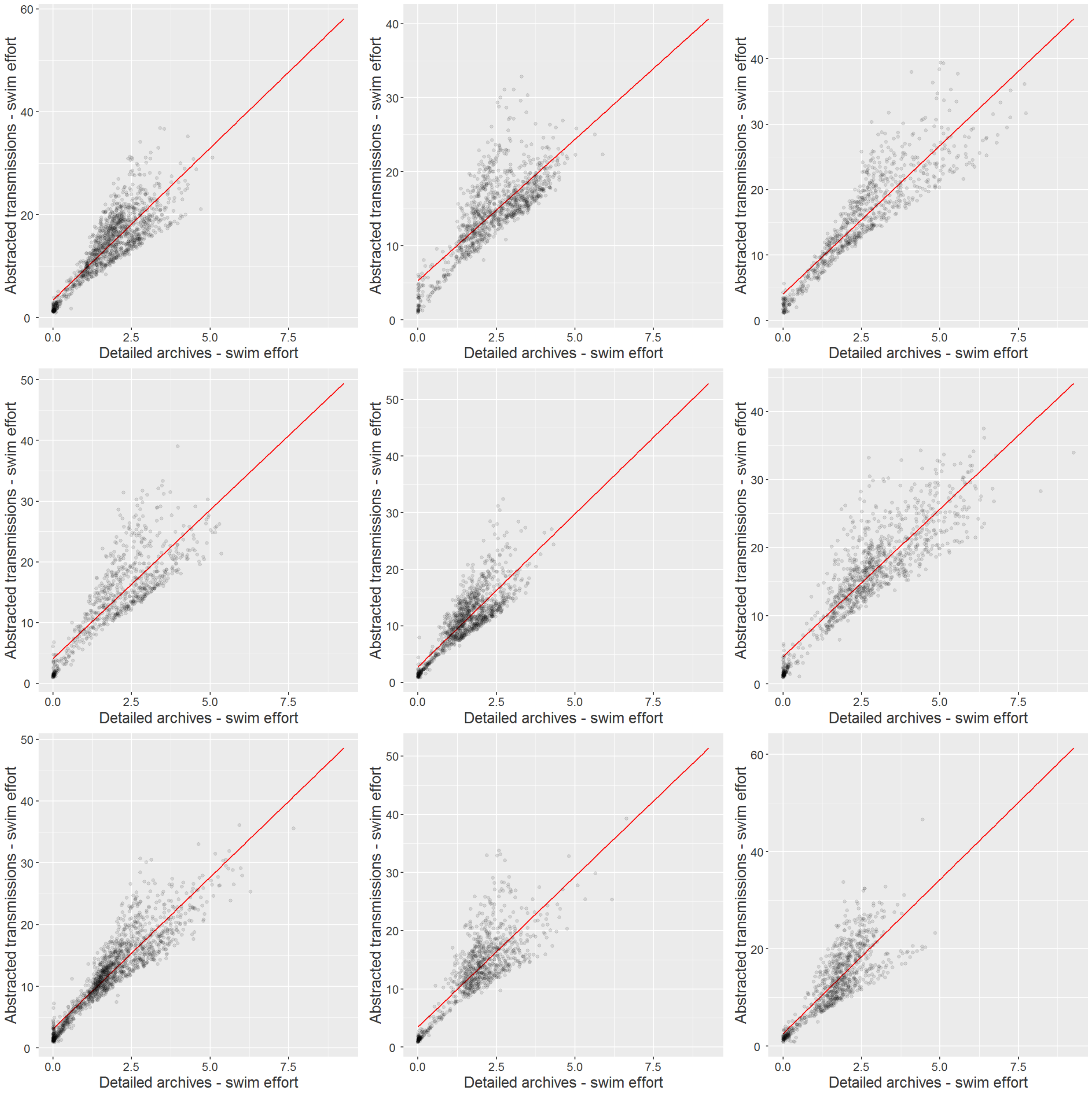

Figure S10. Correlations by individual between swimming effort estimations for bottom phases from archival and transmitted datasets.

S.10 Algorithm improvements for the on-board estimation of swimming effort based upon retrieved archival data from juveniles

Across all dive phases, transmitted swimming efforts were positively biased to those from archival data in a manner that increased with the swimming effort measurement itself (i.e. small transmitted swimming efforts were less positively biased than large values). This overestimation likely reflected the summing of all stroke associated (y-axis) accelerations in on-board processing, rather than the isolation and averaging of individual amplitudes and rates (as in archival processing). Moreover, during bottom phases, the variance of positive bias in transmitted estimates increased with swimming effort (a trend that was consistent across all individuals; see section S.7 of this document), suggesting other movements (e.g. rolling, turning and rapid head jerks) were included in calculations and not sufficiently removed by the filtering process applied during on-board processing. Indeed, whilst descent and ascent phases typically involve directed swimming behaviours to and from prey patches at depth, foraging activity is known to be most concentrated during the bottom phase of a dive increasing the likelihood of the inclusion of these behaviours in swimming effort calculations (Heerah *et al.* 2014). This may be due to the values of the lower limit of the band pass filter (which was based on data from adult southern elephant seals) alongside the omission of an upper limit. Using the archival data from the nine juveniles that returned to the colony, the on-board processing algorithm and its associated thresholds could be altered to improve performance in future applications. Based on power spectral density curves of the nine juveniles from whom archival data was retrieved (Figure S2), future deployments could implement a band pass filter with an upper limit of 2.05Hz to omit rapid movements associated with PrCA behaviours. Whilst keeping the lower limit of the band pass filter to that used in adults would allow for a shift in stroke frequencies as individuals mature, which may be particularly important for longer deployments (e.g. in excess of a year), we found that the performance of the algorithm was greatly improved with a lower limit of 0.49Hz (Figure S11).

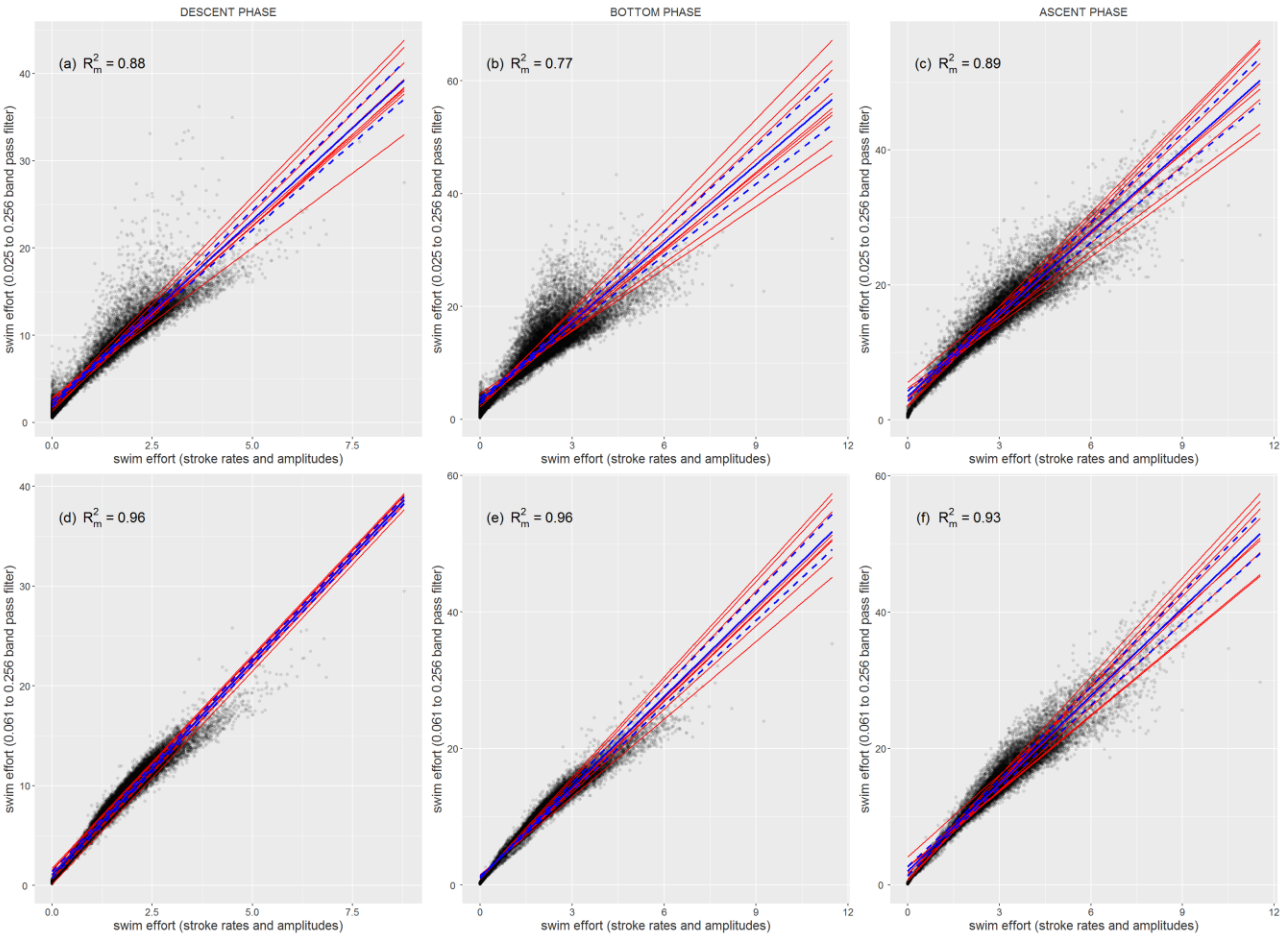

Figure S11. The top row (a:c) shows correlations between swimming effort calculated by isolating individual stroke amplitudes and rates (see methods section of the main article) and those using the on-board processing techniques outlined in the materials and methods of the main article but with a band pass filter of 0.2Hz to 2.05Hz. The bottom row (d:f) shows correlations between swimming effort calculated by isolating individual stroke amplitudes and rates (see methods section of the main article) and those using the on-board processing techniques outlined in the methods section of the main article but with a band pass filter of 0.49Hz to 2.05Hz. Each column corresponds to descent (a&d), bottom (b&e) and ascent (c&f) dive phases. On-board processing techniques using an algorithm with a band pass filter of 0.49Hz to 2.05Hz (corresponding to bottom row plots; d:f) appear to be more closely matched to estimates generated through the isolation of stroke amplitudes and rates. This is backed up by marginal R^2 values (calculated as described in the methods section of the main article and indicated in each subplot) of between 0.93 and 0.96, compared to between 0.77 and 0.89 when the lower threshold of the band pass filter is decreased (corresponding to top row plots; a:c). In all plots, red lines show predicted individual outputs from linear mixed effects models with a random intercept and (barring subplot d due to convergence problems) a random slope. Blues lines show the population mean and dashed blue lines the corresponding bootstrapped 95% confidence intervals.

S.11 Comparisons between pitches from transmitted and archival data excluding dives with ascent and descent phases comprising more than one segment.

Table S3. Modelling results from linear mixed effects models used to assess correlations between abstracted (response) and archival (explanatory) estimates of pitch from only those dives where descent and ascent phases comprise only one segment. Intercept and slope values close to 0 and 1 respectively reflect stronger correlations/likeness between the two data types than those far from 0 and 1. Intercept and slope standard deviation values of each model's random component (R. intercept and R. slope) are reported to evaluate inter-individual differences in algorithm performances. Pseudo R^2 values show the amount of variation accounted for by the fixed component of the model (marginal R^2; R^2_m) and the amount of variation accounted for by random component of the model (R^2_c (conditional R^2) - R^2_m). Root mean square errors are shown for the fitted values of the model versus the retrieved archival data ($RMSE_{mod}$), and the transmitted estimates versus retrieved archival data ($RMSE_{data}$; standardised out with brackets and raw within brackets).

	Intercept	Slope	R. intercept	R. slope	R^2_m	R^2_c- R^2_m	$RMSE_{mod}$	$RMSE_{data}$
Descent phase	-0.12	0.84	0.08	0.09	0.809	0.011	0.05	0.09 (4.67°)
Bottom phase	-	-	-	-	-	-	-	-
Ascent phase	0.08	0.90	0.03	0.03	0.923	0.003	0.19	0.10 (5.07°)

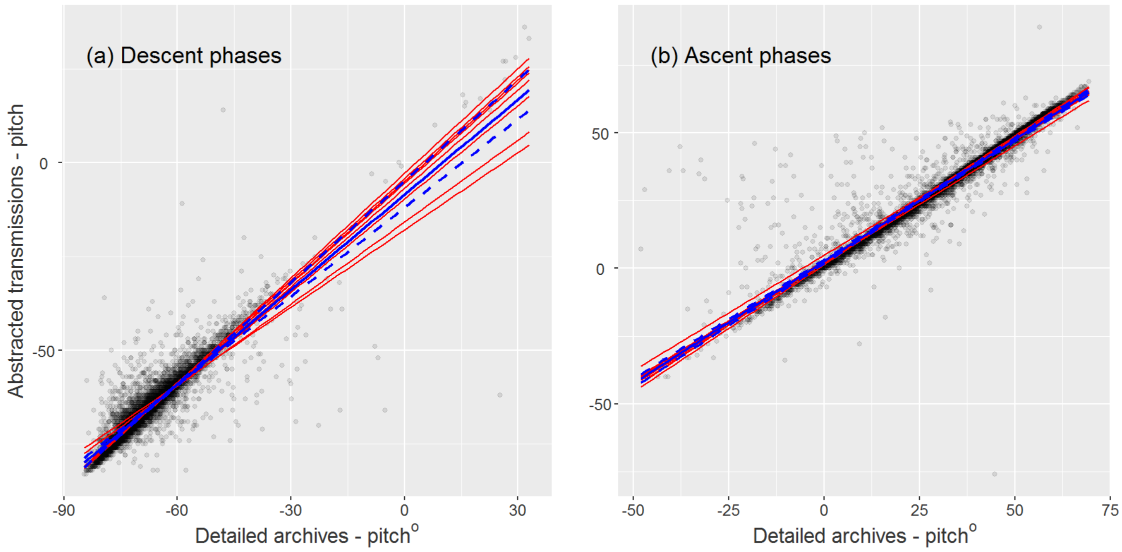

Figure S12. Comparisons between pitch outputs from abstracted transmissions and detailed archives, excluding those dives with descent or ascent phases comprising more than one segment. Each red line represents the intercept slope output for each individual, the blue line the population mean (95% confidence intervals in dashed blue lines), and black points the raw data. From left to right: (a) descent phases and (b) ascent phases.

S.12 References

Aoki, K., Watanabe, Y.Y., Crocker, D.E., Robinson, P.W., Biuw, M., Costa, D.P., Miyazaki, N., Fedak, M.A. & Miller, P.J.O. (2011). Northern elephant seals adjust gliding and stroking patterns with changes in buoyancy: validation of at-sea metrics of body density. *The Journal of Experimental Biology*, **214**, 2973–2987.

Carroll, G., Slip, D., Jonsen, I. & Harcourt, R. (2014). Supervised accelerometry analysis can identify prey capture by penguins at sea. *The Journal of Experimental Biology*, **217**, 4295–4302.

Chimienti, M., Cornulier, T., Owen, E., Bolton, M., Davies, I.M., Travis, J.M.J. & Scott, B.E. (2016). The use of an unsupervised learning approach for characterizing latent behaviours in accelerometer data. *Ecology and Evolution*, **6**, 727–741.

Fahlman, A., Wilson, R., Svard, C., Rosen, D.A.S. & Trites, A.W. (2008). Activity and diving metabolism correlate in Steller sea lion *Eumetopias jubatus*. *Aquatic Biology*, **2**, 75–84.

Gleiss, A.C., Wilson, R.P. & Shepard, E.L.C. (2011). Making overall dynamic body acceleration work: on the theory of acceleration as a proxy for energy expenditure. *Methods in Ecology and Evolution*, **2**, 23–33.

Guinet, C., Vacquie-Garcia, J., Picard, B., Bessigneul, G., Le Bras, Y., Dragon, A.C., Viviant, M., Arnould, J.P.Y. & Bailleul, F. (2014). Southern elephant seal foraging success in relation to temperature and light conditions: insight into prey distribution. *Marine Ecology Progress Series*, **499**, 285–301.

Heerah, K., Hindell, M., Guinet, C. & Charrassin, J.B. (2014). A new method to quantify within dive foraging behaviour in marine predators. *PLoS ONE*, **9**, e99329.

Jeanniard-du-Dot, T., Guinet, C., Arnould, J.P.Y., Speakman, J.R. & Trites, A.W. (2016). Accelerometers can measure total and activity-specific energy expenditures in free-ranging marine mammals only if linked to time activity budgets. *Functional Ecology*.

Jouma'a, J., Le Bras, Y., Richard, G., Vacquie-Garcia, J., Picard, B., Ksabi, N.E. & Guinet, C. (2016). Adjustment of diving behaviour with prey encounters and body condition in a deep diving predator: the Southern Elephant Seal. *Functional Ecology*, **30**, 636–648.

Lavielle, M. (1999). Detection of multiple changes in a sequence of dependent variables. *Stochastic Processes and their Applications*, **83**, 79–102.

Richard, G., Vacquie-Garcia, J., Jouma'a, J., Picard, B., Génin, A., Arnould, J.P.Y., Bailleul, F. & Guinet, C. (2014). Variation in body condition during the post-moult foraging trip of southern elephant seals and its consequences on diving behaviour. *The Journal of Experimental Biology*, **217**, 2609–2619.

Sato, K., Mitani, Y., Cameron, M.F., Siniff, D.B. & Naito, Y. (2003). Factors affecting stroking patterns and body angle in diving Weddell seals under natural conditions. *The Journal of Experimental Biology*, **206**, 1461–1470.

Van der Wal, J., Falconi, L., Januchowski, S., Shoo, L. & Storlie, C. (2014). *SDMTools*. R package

Viviant, M., Jeanniard-du-Dot, T., Monestiez, P., Authier, M. & Guinet, C. (2016). Bottom time does not always predict prey encounter rate in Antarctic fur seals. *Functional Ecology*.

Viviant, M., Trites, A.W., Rosen, D.A.S., Monestiez, P. & Guinet, C. (2010). Prey capture attempts can be detected in Steller sea lions and other marine predators using accelerometers. *Polar Biology*, **33**, 713–719.

Volpov, B.L., Hoskins, A.J., Battaile, B.C., Viviant, M., Wheatley, K.E., Marshall, G., Abernathy, K. & Arnould, J.P.Y. (2015). Identification of prey captures in Australian fur seals (*Arctocephalus pusillus dorifeus*) using head-mounted accelerometers: field validation with animal-born video cameras. *PLoS ONE*, **10**, e0128789.

Wilson, R.P., White, C.R., Quintana, F., Halsey, L.G., Liebsch, N., Martin, G.R. & Butler, P.J. (2006). Moving towards acceleration for estimates of activity-specific metabolic rate in free-living animals: the case of the cormorant. *Journal of Animal Ecology,* **75**, 1081–1090.

Ydesen, K.S., Wisniewska, D.M., Hansen, J.D., Beedholm, K., Johnson, M. & Madsen, P.T. (2014). What a jerk: prey engulfment revealed by high-rate, super-cranial accelerometry on a harbour seal (*Phoca vituline*). *The Journal of Experimental Biology*, **217**, 2239–2243.

Zuur, A.F., Ieno, E.N. & Elphick, C.S. (2010). A protocol for data exploration to avoid common statistical problems. *Methods in Ecology and Evolution*, **1**, 3–14.
